# Polygenic adaptation: From sweeps to subtle frequency shifts

**DOI:** 10.1371/journal.pgen.1008035

**Published:** 2019-03-20

**Authors:** Ilse Höllinger, Pleuni S. Pennings, Joachim Hermisson

**Affiliations:** 1 Mathematics and BioSciences Group, Faculty of Mathematics and Max F. Perutz Laboratories, University of Vienna, Vienna, Austria; 2 Vienna Graduate School of Population Genetics, University of Vienna, Vienna, Austria; 3 Department of Biology, San Francisco State University, San Francisco, California, USA; University of Rochester, UNITED STATES

## Abstract

Evolutionary theory has produced two conflicting paradigms for the adaptation of a polygenic trait. While population genetics views adaptation as a sequence of selective sweeps at single loci underlying the trait, quantitative genetics posits a collective response, where phenotypic adaptation results from subtle allele frequency shifts at many loci. Yet, a synthesis of these views is largely missing and the population genetic factors that favor each scenario are not well understood. Here, we study the architecture of adaptation of a binary polygenic trait (such as resistance) with negative epistasis among the loci of its basis. The genetic structure of this trait allows for a full range of potential architectures of adaptation, ranging from sweeps to small frequency shifts. By combining computer simulations and a newly devised analytical framework based on Yule branching processes, we gain a detailed understanding of the adaptation dynamics for this trait. Our key analytical result is an expression for the joint distribution of mutant alleles at the end of the adaptive phase. This distribution characterizes the polygenic pattern of adaptation at the underlying genotype when phenotypic adaptation has been accomplished. We find that a single compound parameter, the population-scaled background mutation rate Θ_*bg*_, explains the main differences among these patterns. For a focal locus, Θ_*bg*_ measures the mutation rate at all redundant loci in its genetic background that offer alternative ways for adaptation. For adaptation starting from mutation-selection-drift balance, we observe different patterns in three parameter regions. Adaptation proceeds by sweeps for small Θ_*bg*_ ≲ 0.1, while small polygenic allele frequency shifts require large Θ_*bg*_ ≳ 100. In the large intermediate regime, we observe a heterogeneous pattern of partial sweeps at several interacting loci.

## Author summary

It is still an open question how complex traits adapt to new selection pressures. While population genetics champions the search for selective sweeps, quantitative genetics proclaims adaptation via small concerted frequency shifts. To date the empirical evidence of clear sweep signals is more scarce than expected, while subtle shifts remain notoriously hard to detect. In the current study we develop a theoretical framework to predict the expected adaptive architecture of a simple polygenic trait, depending on parameters such as mutation rate, effective population size, size of the trait basis, and the available genetic variability at the onset of selection. For a population in mutation-selection-drift balance we find that adaptation proceeds via complete or partial sweeps for a large set of parameter values. We predict adaptation by small frequency shifts for two main cases. First, for traits with a large mutational target size and high levels of genetic redundancy among loci, and second if the starting frequencies of mutant alleles are more homogeneous than expected in mutation-selection-drift equilibrium, e.g. due to population structure or balancing selection.

## Introduction

Rapid phenotypic adaptation of organisms to all kinds of novel environments is ubiquitous and has been described and studied for decades [1, 2]. However, while the macroscopic changes of phenotypic traits are frequently evident, their genetic and genomic underpinnings are much more difficult to resolve. Two independent research traditions, molecular population genetics and quantitative genetics, have coined two opposite views of the adaptive process on the molecular level: adaptation either by selective sweeps or by subtle allele frequency shifts (*sweeps* or *shifts* from here on).

On the one hand, population genetics works bottom-up from the dynamics at single loci, without much focus on the phenotype. The implicit assumption of the sweep scenario is that selection on the trait results in sustained directional selection also on the level of single underlying loci. Consequently, we can observe phenotypic adaptation at the genotypic level, where selection drives allele frequencies at one or several loci from low values to high values. Large allele frequency changes are the hallmark of the sweep scenario. If these frequency changes occur in a short time interval, conspicuous diversity patterns in linked genomic regions emerge: the footprints of hard or soft selective sweeps [3–6].

On the other hand, quantitative genetics envisions phenotypic adaptation top-down, from the vantage point of the trait. At the genetic level, it is perceived as a collective phenomenon that cannot easily be broken down to the contribution of single loci. Indeed, adaptation of a highly polygenic trait can result in a myriad of ways through “infinitesimally” small, correlated changes at the interacting loci of its basis (e.g. [1, 7, 8]. Conceptually, this view rests on the infinitesimal model by Fisher (1918) [9] and its extensions (e.g. [10]). Until a decade ago, the available moderate sample sizes for polymorphism data had strongly limited the statistical detectability of small frequency shifts. Therefore, the detection of sweeps with clear footprints was the major objective for many years. Since recently, however, huge sample sizes (primarily of human data) enable powerful genome-wide association studies (GWAS) to resolve the genomic basis of polygenic traits. Consequently, following conceptual work by Pritchard and coworkers [7, 11], there has been a shift in focus to the detection of polygenic adaptation from subtle genomic signals (e.g. [12–14], reviewed in [15]). Very recently, however, some of the most prominent findings of polygenic adaptation in human height have been challenged [16, 17]. As it turned out, the methods are highly sensitive to confounding effects in GWAS data due to population stratification.

While discussion of the empirical evidence is ongoing, the key objective for theoretical population genetics is to clarify the conditions (mutation rates, selection pressures, genetic architecture) under which each adaptive scenario, sweeps, shifts—or any intermediate type—should be expected in the first place. Yet, the number of models in the literature that allow for a comparison of alternative adaptive scenarios at all is surprisingly limited (see also [18]). Indeed, quantitative genetic studies based on the infinitesimal model or on summaries (moments, cumulants) of the breeding values do not resolve allele frequency changes at individual loci (e.g. [19–22]). In contrast, sweep models with a single locus under selection in the tradition of Maynard Smith and Haigh [3], or models based on adaptive walks or the adaptive dynamics framework (e.g. [23–25]) only allow for adaptive substitutions or sweeps. A notable exception is the pioneering study by Chevin and Hospital [26]. Following Lande [27], these authors model adaptation at a single major quantitative trait locus (QTL) that interacts with an “infinitesimal background” of minor loci, which evolves with fixed genetic variance. Subsequent models [28, 29] trace the allele frequency change at a single QTL in models with 2-8 loci. Still, these articles do not discuss polygenic adaptation patterns. Most recently, Jain and Stephan [30, 31] studied the adaptive process for a quantitative trait under stabilizing selection with explicit genetic basis. Their analytical approach allows for a detailed view of allele frequency changes at all loci without constraining the genetic variance. However, the model is deterministic and thus ignores the effects of genetic drift. Below, we study a polygenic trait that can adapt via sweeps or shifts under the action of all evolutionary forces in a panmictic population (mutation, selection, recombination and drift). Our model allows for comprehensive analytical treatment, leading to a multi-locus, non-equilibrium extension of Wright’s formula [32] for the joint distribution of allele frequencies at the end of the adaptive phase. This way, we obtain predictions concerning the adaptive architecture of polygenic traits and the population genetic variables that delimit the corresponding modes of adaptation.

The article is organized as follows. The Model section motivates our modeling decisions and describes the simulation method. We also give a brief intuitive account of our analytical approach. In the Results part, we describe our findings for a haploid trait with linkage equilibrium among loci. All our main conclusions in the Discussion part are based on the results displayed here. Further model extensions and complications (diploids, linkage, and alternative starting conditions) are relegated to appendices. Finally, we describe our analytical approach and derive all results in a comprehensive Mathematical Appendix (S2 Appendix). For the ease of reading, we have tried to keep both the main text and the Mathematical Appendix independent and largely self-contained.

## Methods

In the current study, we aim for a “minimal model” of a trait that allows us to clarify which evolutionary forces favor sweeps over shifts and vice versa (as well as any intermediate patterns). For shifts, alleles need to be able to hamper the rise of alleles at other loci via negative epistasis for fitness, e.g. diminishing returns epistasis. Indeed, otherwise one would only observe parallel sweeps. Negative fitness epistasis is frequently found in empirical studies (e.g. [33]) and implicit to the Gaussian selection scheme (e.g. [26, 30, 31]). More fundamentally, diminishing returns are a consequence of partial or complete redundancy of genetic effects across loci or gene pathways. Adaptive phenotypes (such as pathogen resistance or a beneficial body coloration) can often be produced in many alternative ways, such that redundancy is a common characteristic of beneficial mutations.

As our basic model, we focus on a haploid population and study adaptation for a polygenic, binary trait with full redundancy of effects at all loci. We assume a non-additive genotype-phenotype map where any single mutation switches the phenotype from its ancestral state (e.g. “non-resistant”) to the adaptive state (“resistant”). Further mutations have no additional effect. On the population level, adaptation can be produced by a single locus where the beneficial allele sweeps to fixation, or by small frequency shifts of alleles at many different loci in different individuals—or any intermediate pattern. The symmetry among loci (no build-in advantage of any particular locus) and complete redundancy of locus effects provides us with a trait architecture that is favorable for collective adaptation via small shifts—and with a modeling framework that allows for analytical treatment. The same model has been used in a preliminary simulation study [6]. In the context of parallel adaptation in a spatially structured population, analogous model assumptions with redundant loci have been used [34–36]. In a second step, we extend our basic model to relax the redundancy condition, as described below.

### Basic model

**Fig 1:**
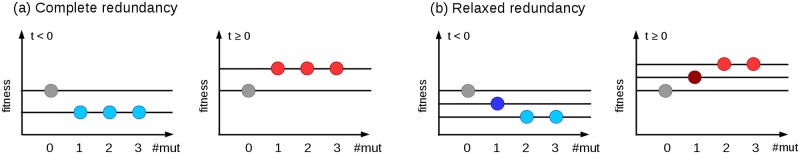
Fitness schemes. The fitness of individuals carrying 0, 1, 2, 3… mutations (y-axis) are given for the complete redundancy (a) and relaxed redundancy (b) model, respectively. Grey balls show the fitness of ancestral wildtype individuals (without mutations). Colored balls represent individuals carrying at least one mutation, for time points *t* < 0 before the environmental change in blue and for *t* ≥ 0 in red.

Consider a panmictic population of *N*_*e*_ haploids, with a binary trait *Z* (with phenotypic states *Z*_0_ “non-resistant” and *Z*_1_ “resistant”, see Fig 1). The trait is governed by a polygenic basis of *L* bi-allelic loci with arbitrary linkage (we treat the case of linkage equilibrium in the main text and analyze the effects of linkage in S1 Appendix, Section A). Only the genotype with the ancestral alleles at all loci produces phenotype *Z*_0_, all other genotypes produce *Z*_1_, irrespective of the number of mutations they carry. Loci mutate at rate *μ*_*i*_, 1 ≤ *i* ≤ *L*, per generation (population mutation rate at the *i*th locus: 2*N*_*e*_ *μ*_*i*_ = Θ_*i*_) from the ancestral to the derived allele. We ignore back mutation. The mutant phenotype *Z*_1_ is deleterious before time *t* = 0, when the population experiences a sudden change in the environment (e.g. arrival of a pathogen). *Z*_1_ is beneficial for time *t* > 0. The Malthusian (logarithmic) fitness function of an individual with phenotype *Z* reads

$$\begin{array}{c} W\left(Z\right)=\{\begin{array}{cc} \;s_{d}Z & \text{for}\;t<0\\ \;s_{b}Z & \text{for}\;t\geq 0. \end{array} \end{array}$$ (1)

Without loss of generality, we can assume *Z*_0_ = 0 and *Z*_1_ = 1. We then have *W*(*Z*_0_) = 0. Furthermore, *W*(*Z*_1_) = *s*_*d*_ < 0, respectively *W*(*Z*_1_) = *s*_*b*_ > 0, measure the strength of directional selection on *Z* (e.g. cost and benefit of resistance) before and after the environmental change. For the basic model, we assume that the population is in mutation-selection-drift equilibrium at time *t* = 0.

### Model extensions

We extend the basic model in several directions. This includes linkage (S1 Appendix, Section A), alternative starting conditions at time *t* = 0 (S1 Appendix, Section B), diploids (S1 Appendix, Section C), and arbitrary time-dependent selection *s*(*t*) (S2 Appendix, Section M.1). Here, we describe how we relax the assumption of complete redundancy of all loci. Diminishing returns epistasis, e.g. due to Michaelis-Menten enzyme kinetics, will frequently not lead to complete adaptation in a single step, but may require multiple steps before the trait optimum is approached. In a model of incomplete redundancy, we thus assume that a first beneficial mutation only leads to partial adaptation. We thus have three states of the trait, the ancestral state for the genotype without mutations, *Z*_0_ = 0 (non-resistant), a phenotype *Z*_*δ*_ = *δ* (partially resistant) for genotypes with a single mutation, and the mutant state *Z*_1_ = 1 (fully resistant) for all genotypes with at least two mutations, see Fig 1(b). For diminishing returns epistasis, we require $\frac{1}{2}\leq \delta <1$. The fitness function is as in Eq (1). A model with asymmetries in the single-locus effects is discussed in S1 Appendix, Section D.

### Simulation model

For the models described above, we use Wright-Fisher simulations for a haploid, panmictic population of size *N*_*e*_, assuming linkage equilibrium between all *L* loci in discrete time. Selection and drift are implemented by independent weighted sampling based on the marginal fitnesses of the ancestral and mutant alleles at each locus. Due to linkage equilibrium, the marginal fitnesses only depend on the allele frequencies and not genotypes. Ancestral alleles mutate with probability *μ*_*i*_ per generation at locus *i*. We start our simulations with a population that is monomorphic for the ancestral allele at all loci. The population evolves for 8*N*_*e*_ generations under mutation and deleterious selection to reach (approximate) mutation-selection-drift equilibrium. Following [6, 37], we condition on adaptation from the ancestral state and discard all runs where the deleterious mutant allele (at any locus) reaches fixation during this time. (We do not show results for cases with very high mutation rates and weak deleterious selection when most runs are discarded). At the time of environmental change, selection switches from negative to positive and simulation runs are continued until a prescribed stopping condition is reached.

We are interested in the genetic architecture of adaptation—the joint distribution of mutant frequencies across all loci—at the end of the rapid adaptive phase. Following [31], we define this phase as “the time until the phenotypic mean reaches a value close to the new optimum”. Specifically, we stop simulations when the mean fitness $\bar{W}$ in the population has increased up to a proportion *f*_*w*_ of the maximal attainable increase from the ancestral to the derived state,

$$\begin{array}{c} \frac{W\left(Z_{1}\right)-\bar{W}}{W\left(Z_{1}\right)-W\left(Z_{0}\right)}=f_{w}\;. \end{array}$$ (2)

For the basic model with complete redundancy, this simply corresponds to a residual proportion *f*_*w*_ of individuals with ancestral phenotype in the population. Extensions of the simulation scheme to include linkage or diploid individuals are described in S1 Appendix, Sections A and C.

*Parameter choices*: Unless explicitly stated otherwise, we simulate *N*_*e*_ = 10 000 individuals, with beneficial selection coefficients *s*_*b*_ = 0.1 and 0.01, combined with deleterious selection coefficients *s*_*d*_ = −0.1 and *s*_*d*_ = −0.001 for low and high levels of SGV, respectively. (The corresponding Wrightian fitness values used as sampling weights in discrete time are 1 + *s*_*b*_ and 1 + *s*_*d*_.) We investigate *L* = 2 to 100 loci. We usually (except in S1 Appendix, Section D) assume equal mutation rates at all loci, *μ*_*i*_ = *μ* and define Θ_*l*_ = 2*N*_*e*_ *μ* as the locus mutation parameter. Mutation rates are chosen such that Θ_*bg*_ ≔ 2*N*_*e*_ *μ*(*L* − 1) (the background mutation rate, formally defined below in Eq (10)) takes values from 0.01 to 100. We typically simulate 10 000 replicates per mutation rate and stop simulations when the population has reached the new fitness optimum up to *f*_*w*_ = 0.05. In the model with complete redundancy, we thus stop simulations when the frequency of individuals with mutant phenotype *Z*_1_ has increased to 95%. Different stopping conditions are explored in S1 Appendix, Section G.

### Analytical analysis

**Fig 2:**
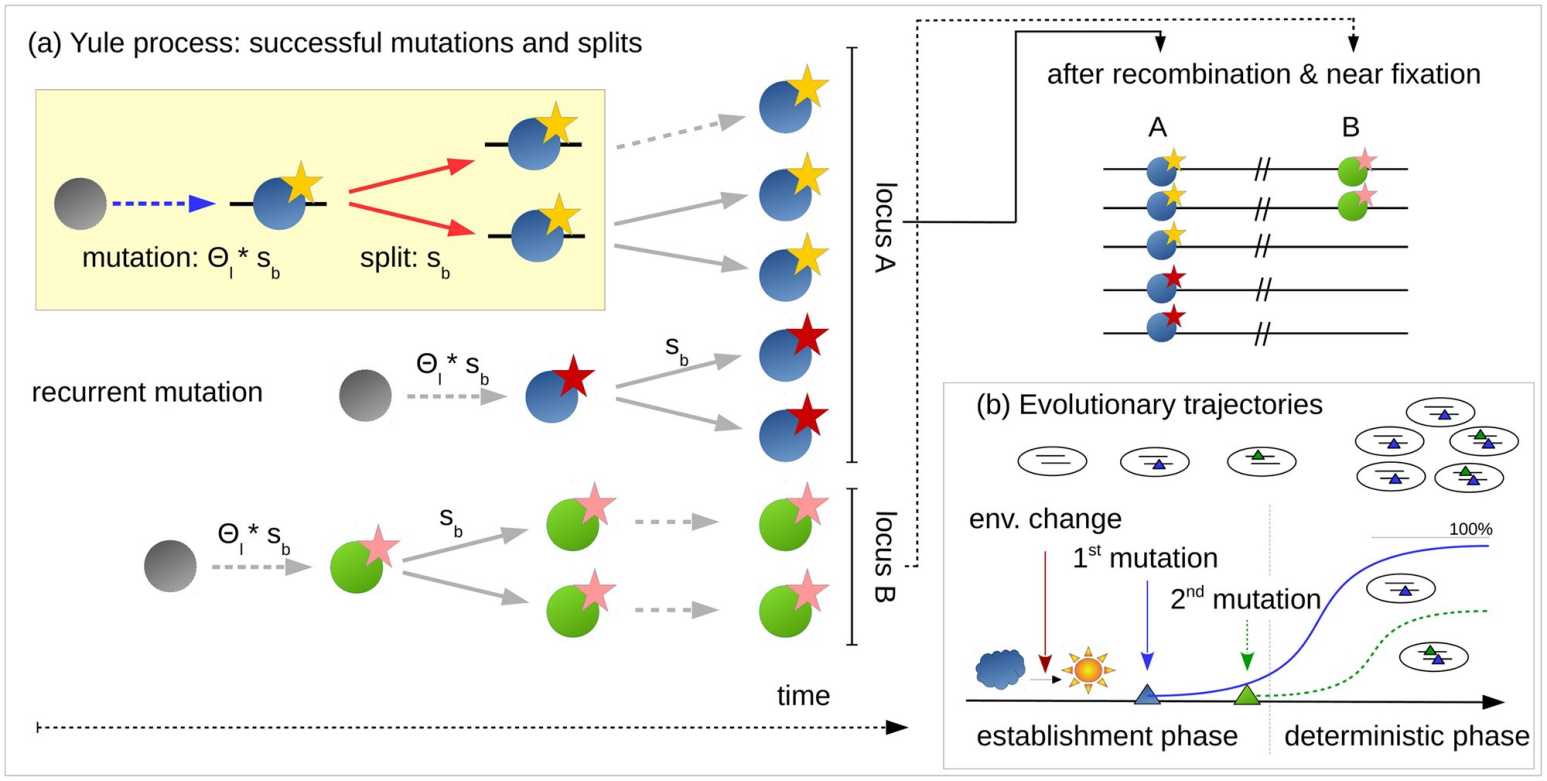
Phases of polygenic adaptation. The adaptive process is partitioned into two phases. The initial, stochastic phase describes the establishment of mutant alleles. Ignoring epistasis during this phase, it can be described by a *Yule* process (panel a), with two types of events (yellow box). Either a new mutation occurs and establishes with rate Θ_*l*_ ⋅ *s*_*b*_ or an existing mutant line splits into two daughter lines at rate *s*_*b*_. Mutations and splits can occur in parallel at all loci of the polygenic basis, (here 2 loci, shown in green and blue). Yellow and red stars at the blue locus indicate establishment of two redundant mutations at this locus. When mutants have grown to higher frequencies, the adaptive process enters its second, deterministic phase, where drift can be ignored (panel b). During the deterministic phase, the trajectories of mutations at different loci constrain each other due to epistasis. We refer to the locus ending up at the highest frequency as the *major* locus (here in blue) and to all others as *minor* loci (here one in green).

We partition the adaptive process into two phases (see Fig 2 for illustration). An initial *stochastic phase*, governed by selection, drift, and mutation describes the origin and establishment of mutant alleles at all loci. We call mutants “established” if they are not lost again due to genetic drift. The subsequent *deterministic phase* governs the further evolution of established alleles until the stopping condition is reached as described above. While mutation and drift can be ignored during the deterministic phase, interaction effects due to epistasis and linkage become important (in our model, they enter, in particular, through the stopping condition). We give a brief overview of our analytical approach below; parameters are summarized in Table 1. A detailed account with the derivation of all results is provided in the Mathematical Appendix S2 Appendix.

**Table 1:** Glossary.

*L*	…	size of polygenic basis (no. of loci)
*s*_*d*_, *s*_*b*_	…	selection coefficient before/after the environment changes
$p_{i}≔\frac{k_{i}}{N}$	…	mutant allele frequency at locus *i*
$x_{i}≔\frac{k_{i}}{k_{1}}=\frac{p_{i}}{p_{1}}$	…	mutant allele frequency ratio: locus *i* / locus 1
*f*_*w*_	…	frequency of ancestral phenotype
*μ*_*i*_	…	allelic mutation rate at locus *i*
Θ_*i*_ = 2*N*_*e*_ *μ*_*i*_	…	haploid population mutation rate at locus *i*
**Θ** = {Θ_1_, …, Θ_*L*_}	…	vector of all locus population mutation rates
Θ_*l*_	…	locus pop. mut. rate, for model with equal mutation rates
Θ_*bg*_	…	background mutation rate, Eq (10)
$B\left[\Theta \right]=\frac{\prod_{i\geq 1}\Gamma \left(\Theta_{i}\right)}{\sum_{i\geq 1}\Gamma \left(\Theta_{i}\right)}$	…	Beta function, where Γ(Θ_*i*_) is the Gamma function

During the *stochastic phase*, we model the origin and spread of mutant copies as a so-called *Yule pure birth process* following [38] and [39]. The idea of this approach is that we only need to keep track of mutations that found “immortal lineages”, i.e. derived alleles that still have surviving offspring at the time of observation (see Fig 2 for the case of *L* = 2 loci). Forward in time, new immortal lineages can be created by two types of events: new mutations at all loci start new lineages, while birth events lead to splits of existing lineages into two immortal lineages. For *t* > 0 (after the environmental change), in particular, new mutations at the *i*th locus arise at rate *N*_*e*_*μ*_*i*_ per generation and are destined to become established in the population with probability ≈ 2*s*_*b*_. Similarly, birth of new immortal lineages due to split events in the Yule process occur at rate *s*_*b*_ (because the selection coefficient measures the excess of births over deaths in the underlying population). For the origin of new immortal lineages in the Yule process and their subsequent splitting we thus obtain the rates

$$\begin{array}{c} p_{\text{mut},i}\approx N_{e}\mu_{i}\cdot 2s_{b}=\Theta_{i}s_{b}\;;\;p_{\text{split}}\approx s_{b}. \end{array}$$ (3)

Extended results including standing genetic variation and time-dependent fitness are given in the Appendix. Assume now that there are currently {*k*_1_, …*k*_*L*_}, 0 ≤ *k*_*j*_ ≪ *N*_*e*_ mutant lineages at the *L* loci. The probability that the next event (which can be a split or a mutation) occurs at locus *i* is

$$\begin{array}{c} \frac{k_{i}\cdot p_{\text{split}}+p_{\text{mut},i}}{\sum_{j=1}^{L}\left(k_{j}\cdot p_{\text{split}}+p_{\text{mut},j}\right)}=\frac{k_{i}+\Theta_{i}}{\sum_{j=1}^{L}\left(k_{j}+\Theta_{j}\right)}. \end{array}$$ (4)

Importantly, all these transition probabilities among states of the Yule process are constant in time and independent of the mutant fitness *s*_*b*_, which cancels in the ratio of the rates. As the number of lineages at all loci increases, their joint distribution (across replicate realizations of the Yule process) approaches a limit. In particular, as shown in the Appendix, the joint distribution of frequency ratios *x*_*i*_ ≔ *k*_*i*_/*k*_1_ in the limit *k*_1_ → ∞ is given by an *inverted Dirichlet distribution*

$$\begin{array}{c} {\text{P}}_{\text{inDir}}\left[x|\Theta \right]=\frac{1}{B[\Theta ]}\prod_{j=2}^{L}x_{j}^{\Theta_{j}-1}(1+\sum_{i=2}^{L}x_{i}{)}^{-\sum_{i=1}^{L}\Theta_{i}} \end{array}$$ (5)

where **x** = (*x*_2_, …, *x*_*L*_) and **Θ** = (Θ_1_, …, Θ_*L*_) are vectors of frequency ratios and locus mutation rates, respectively, and where $B\left[\Theta \right]=\frac{\prod_{j=1}^{L}\Gamma \left(\Theta_{j}\right)}{\sum_{j=1}^{L}\Gamma \left(\Theta_{j}\right)}$ is the generalized Beta function and Γ(*z*) is the Gamma function. Note that Eq (5) depends only on the locus mutation rates, but not on selection strength.

After the initial stochastic phase, the dynamics of established mutant lineages that have evaded stochastic loss can be adequately described by deterministic selection equations. For allele frequencies *p*_*i*_ at locus *i*, assuming linkage equilibrium, we obtain (consult S2 Appendix, Section M.1, Eq (M.2a), for a detailed derivation)

$$\begin{array}{c} {\dot{p}}_{i}=p_{i}\left(W\left(Z_{1}\right)-\bar{W}\right)=s_{b}p_{i}\left(Z_{1}-\bar{Z}\right), \end{array}$$ (6)

where $\bar{W}$ and $\bar{Z}$ are population mean fitness and mean trait value. For the mutant frequency ratios *x*_*i*_ = *p*_*i*_/*p*_1_, we obtain

$$\begin{array}{c} {\dot{x}}_{i}=\frac{d}{dt}(\frac{p_{i}}{p_{1}})=\frac{{\dot{p}}_{i}p_{1}-p_{i}{\dot{p}}_{1}}{p_{1}^{2}}=0\;. \end{array}$$ (7)

We thus conclude that the frequency ratios *x*_*i*_ do not change during the deterministic phase. In particular, this means that Eq (5) still holds at our time of observation at the end of the rapid adaptive phase. This is even true with linked loci. Finally, derivation of the joint distribution of mutant frequencies *p*_*i*_ (instead of frequency ratios *x*_*i*_) at the time of observation requires a transformation of the density. In general, this transformation depends on the stopping condition *f*_*w*_ and on other factors such as linkage. Assuming linkage equilibrium among all selected loci, we obtain (see S2 Appendix, Theorem 2, Eq (M.20))

$$\begin{array}{c} {\text{P}}_{f_{w}}\left[p|\Theta \right]=\frac{\delta_{\prod_{j=1}^{L}\left(1-p_{j}\right)-f_{w}}}{B[\Theta ]}\prod_{j=1}^{L}p_{j}^{\Theta_{j}-1}(\sum_{i=1}^{L}p_{i}{)}^{-\sum_{i=1}^{L}\Theta_{i}}(\sum_{j=1}^{L}\frac{f_{w}p_{j}}{1-p_{j}}) \end{array}$$ (8)

for **p** = (*p*_1_, …, *p*_*L*_) in the *L*-dimensional hypercube of allele frequencies. The delta function *δ*_*X*_ restricts the distribution to the *L* − 1 dimensional manifold defined via the stopping condition $f_{w}=\prod_{j=1}^{L}\left(1-p_{j}\right)$. Further expressions, also including linkage, are given in S2 Appendix and in S1 Appendix, Section A. In general, the joint distribution corresponds to a family of generalized Dirichlet distributions.

We assess the adaptive architecture not as a function of time, but as a function of progress in phenotypic adaptation, measured by *f*_*w*_, Eq (2). Hence, *f*_*w*_ rather than time *t* plays the role of a dynamical variable in the joint distribution, see Eq (8). In the special case *f*_*w*_ → 0 (i.e. complete adaptation, enforcing fixation at at least one locus), this distribution is restricted to a boundary face of the allele frequency hypercube and Eq (8) reduces to the inverted Dirichlet distribution given above in Eq (5). In the Results section below, we assess our analytical approximations for the joint distributions of adaptive alleles, Eqs (5) and (8), and discuss their implications in the context of scenarios of polygenic adaptation, ranging from sweeps to small frequency shifts.

## Results

While the joint distribution of allele frequencies, Eq (8), provides comprehensive information of the adaptive architecture, low-dimensional summary statistics of this distribution are needed to describe and classify distinct types of polygenic adaptation. To this end, we order loci according to their contribution to the adaptive response. In particular, we call the locus with the highest allele frequency at the stopping condition the *major locus* and all other loci *minor loci*. Minor loci are further ordered according to their frequency (first minor, second minor, etc.). The marginal distributions of the major locus or *k*th minor locus are 1-dimensional summaries of the joint distribution. Importantly, these summaries are still *collective* because the role of any specific locus (its order) is defined through the allele frequencies at *all* loci. This is different for the marginal distribution at a fixed focal locus, which is chosen irrespective of its role in the adaptive process, e.g. [26, 28, 29].

Concerning our nomenclature, note that the *major* and *minor* loci do not differ in their effect size, as they are completely redundant. Still, the major locus is the one with the largest contribution to the adaptive response and would yield the strongest association in a GWAS case-control study.

In the following, we analyze adaptive trait architectures in three steps. In the Section *Expected allele frequency ratio*, we use the expected allele frequency ratio of minor and major loci as a one-dimensional summary statistic. Subsequently, in Section *Genomic architecture of polygenic adaptation*, we analyze the marginal distributions of major and minor loci for a trait with 2 to 100 loci. Finally, in Section *Relaxing complete redundancy*, we investigate the robustness of our results under conditions of relaxed redundancy. Further results devoted to diploids, linkage, asymmetric loci, and alternative starting conditions are provided in S1 Appendix.

### Expected allele frequency ratio

For our biological question concerning the type of polygenic adaptation, the ratio of allele frequency changes of minor over major loci is particularly useful. With “sweeps at few loci”, we expect large differences among loci, resulting in ratios that deviate strongly from 1. In contrast, with “subtle shifts at many loci”, multiple loci contribute similarly to the adaptive response and ratios should range close to 1. Our theory (explained above) predicts that these ratios are the outcome of the stochastic phase, and their distribution is preserved during the deterministic phase. They are thus independent of the precise time of observation. For our results in this section, we assume that the mutation rate at all *L* loci is equal, Θ_*i*_ ≡ Θ_*l*_, for all 1 ≤ *i* ≤ *L*. This corresponds to the symmetric case that is most favorable for a “small shift” scenario. Results for asymmtric mutation rates are reported in Appendix S1 Appendix, Section D.

Consider first the case of *L* = 2 loci. There is then a single allele frequency ratio “minor over major locus”, which we denote by *x*. For two loci, the joint distribution of frequency ratios from Eq (5) reduces to a *beta-prime* distribution. Conditioning on the case that the first locus is the major locus (probability 1/2 for the symmetric model), we obtain for 0 ≤ *x* ≤ 1,

$$\begin{array}{c} P_{\beta^{\prime }}\left[x|\Theta_{l}\right]=\frac{2\Gamma (2\Theta_{l})}{{\left(\Gamma \left(\Theta_{l}\right)\right)}^{2}}\;x^{\Theta_{l}-1}{\left(1+x\right)}^{-2\Theta_{l}}, \end{array}$$ (9)

**Fig 3:**
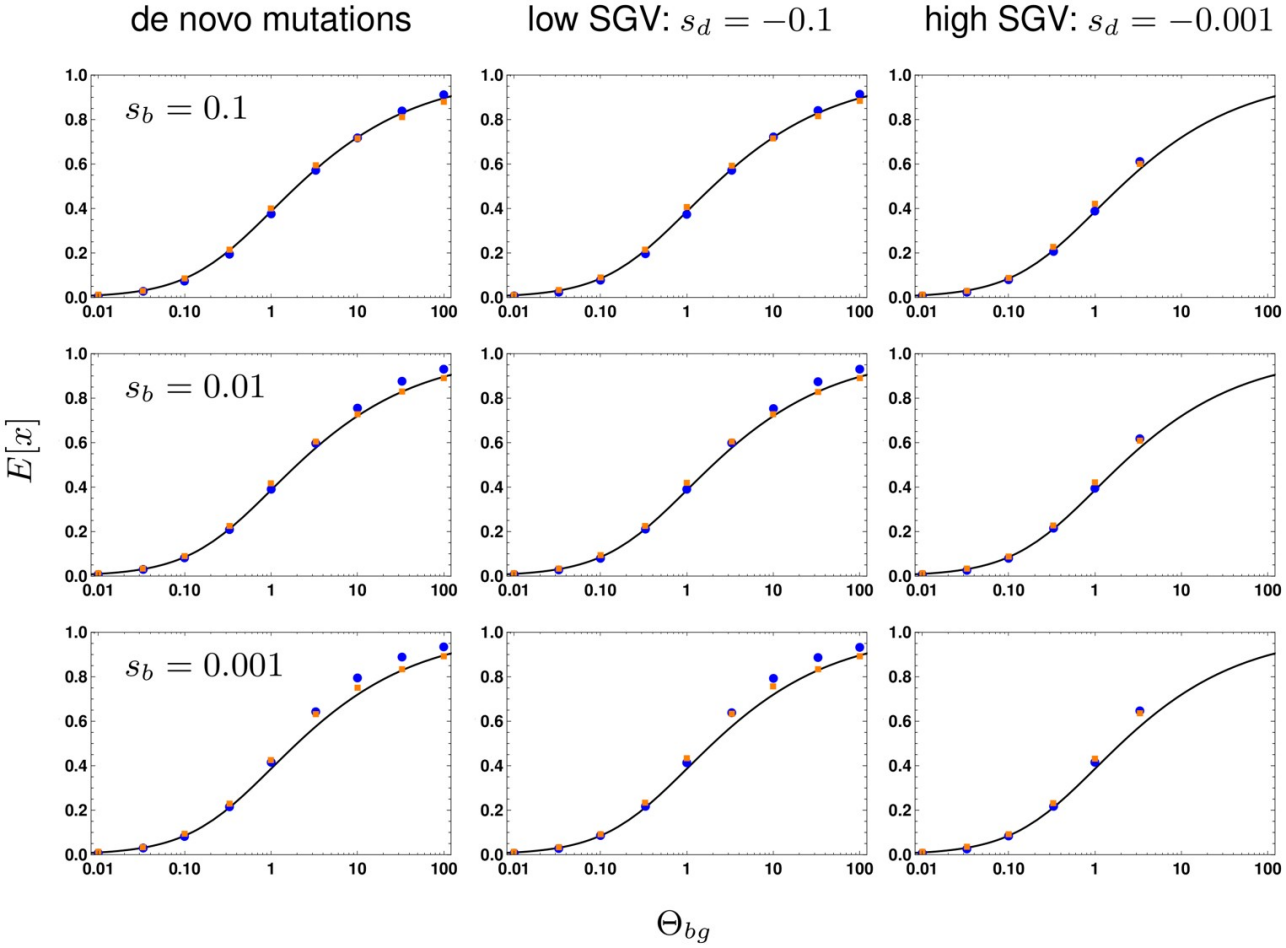
Effect of selection strength and SGV on the frequency ratio E[*x*]. We contrast the expected allele frequency ratios of the first minor locus (with the second highest frequency) over the major locus (with the highest frequency) for 2 loci (blue dots) and for 10 loci (orange dots) with analytical predictions (Appendix, Eq M.16, black curve). E[*x*] is shown as a function of Θ_*bg*_ (= Θ_*l*_ for the 2-locus case). Panels correspond to different strengths of positive selection (*s*_*b*_, rows) and levels of SGV (no SGV, strongly deleterious *s*_*d*_ = −0.1, weakly deleterious *s*_*d*_ = −0.001, columns). We find that neither factor alters the expected ratio. We do not obtain results for Θ_*bg*_ ≥ 10 and *s*_*d*_ = −0.001, where strong recurrent mutation overwhelmes weak selection, such that mutant alleles fix even before the environmental change. Results for 10 000 replicates, standard errors < 0.005 (smaller than symbols).

Fig 3 compares the expectation of this analytical prediction with simulation results for a range of parameters for the strength of beneficial selection *s*_*b*_ and for the level of standing genetic variation (SGV implicitly given by the strength of deleterious selection *s*_*d*_ before the environmental change). There are two main observations. First, the simulation results demonstrate the importance of the scaled mutation rate Θ_*bg*_ ≡ Θ_*l*_ (for two loci). Low Θ_*bg*_ leads to sweep-like adaptation (heterogeneous adaptation response among loci, E[*x*] ≪ 1), whereas high Θ_*bg*_ leads to shift-like adaptation (homogeneous response, E[*x*] near 1). Second, the panels show that the selection intensity given by *s*_*d*_ and *s*_*b*_ has virtually no effect. Both results are predicted by the analytical theory (Eq (9)). In S1 Appendix, Section A, we further show that these results hold for arbitrary degrees of linkage (including complete linkage).

For more than two loci, *L* > 2, one-dimensional marginal distributions of the joint distribution, Eq (5), generally require (*L* − 1)-fold integration, which can be complicated. However, it turns out that the key phenomena to characterize the adaptive architecture can still be captured by the 2-locus formalism, with appropriate rescaling of the mutation rate. For the general *L*-locus model, we broaden our definition of the summary statistic *x* above to describe the allele frequency ratio of the *first minor* locus and the major locus. To relate the distribution of *x* in the *L*-locus model to the one in the 2-locus model, we reason as follows: For small locus mutation rates Θ_*l*_, the order of the loci is largely determined by the order at which mutations that are destined for establishment originate at these loci. *I.e*., the locus where the first mutation originates ends up as the major locus and the first minor locus is usually the second locus where a mutation destined for establishment originates. The distribution of the allele frequency ratio *x* is primarily determined by the distribution of the waiting time for this second mutation after origin of the first mutation at the major locus. In the 2-locus model, this time will be exponentially distributed, with parameter 1/Θ_*l*_. In the *L*-locus model, however, where *L* − 1 loci with total mutation rate Θ_*l*_(*L* − 1) compete for being the “first minor”, the parameter for the waiting-time distribution reduces to 1/(Θ_*l*_(*L* − 1)). We thus see from this argument that the decisive parameter is the cumulative *background mutation rate*

$$\begin{array}{c} \Theta_{bg}=\left(L-1\right)\Theta_{l} \end{array}$$ (10)

at all minor loci in the background of the major locus. In Fig 3 (orange dots) we show simulations of a *L* = 10 locus model with an appropriately rescaled locus mutation rate Θ_*l*_ → Θ_*l*_/9, such that the background rate Θ_*bg*_ is the same as for the 2-locus model. We see that the analytical prediction based on the 2-locus model provides a good fit for the 10-locus model. A more detailed discussion of this type of approximation is given in S1 Appendix, Section F.

### Genomic architecture of polygenic adaptation

While the distribution of allele frequency ratios, Eqs (5) and (9), offers a coarse (but robust) descriptor of the adaptive scenario, the joint distribution of allele frequencies at the end of the adaptive phase, Eq (8), allows for a more refined view. In contrast to the distribution of ratios, the results now depend explicitly on the stopping condition (the time of observation) and on linkage among loci. We assume linkage equilibrium in this section and assess the mutant allele frequencies when the frequency of the remaining wildtype individuals in the population has dropped to a fixed value of *f*_*w*_ = 0.05. In S1 Appendix, Section G, we complement these results and study the changes in the adaptive architecture when *f*_*w*_ is varied.

**Fig 4:**
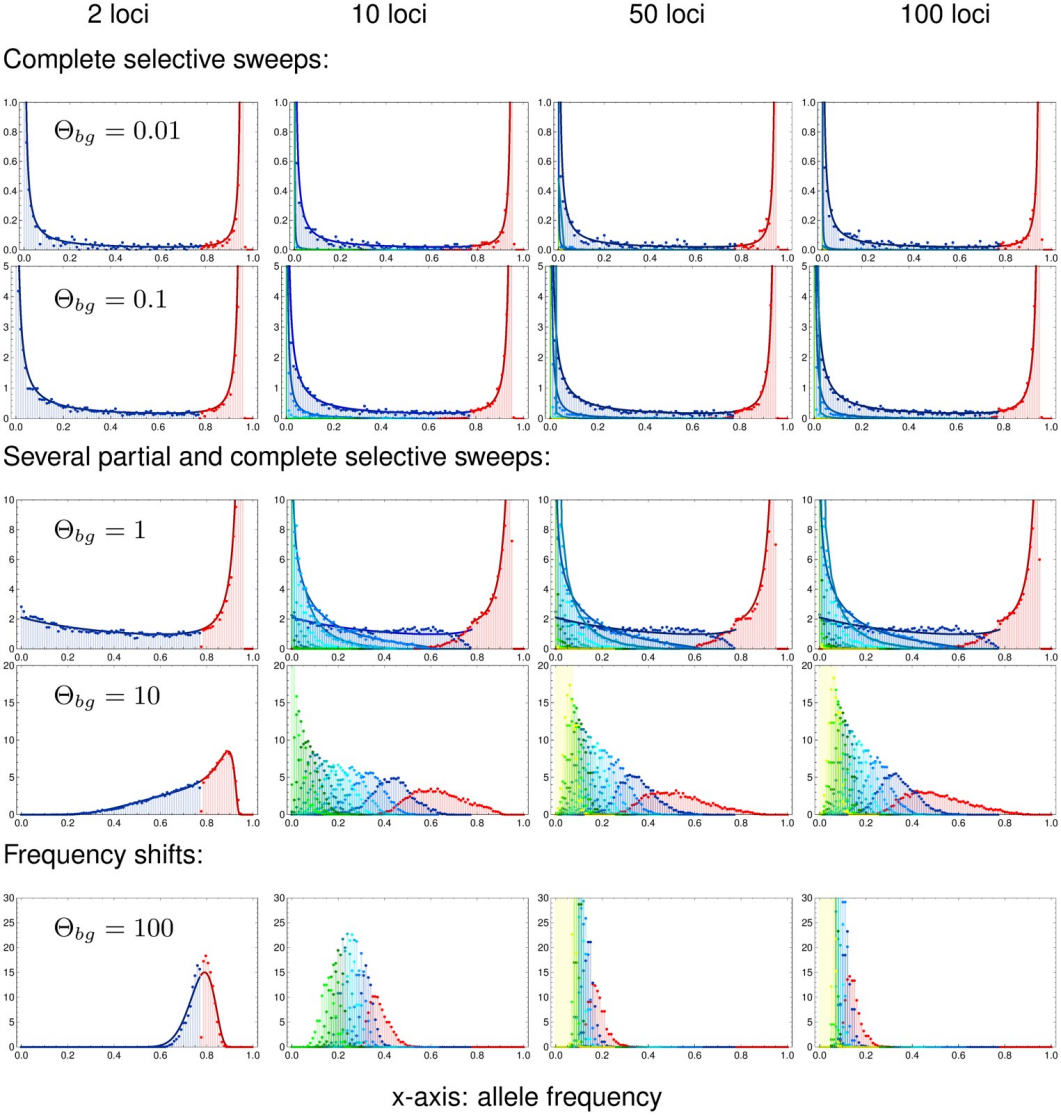
Genomic architecture of polygenic adaptation. We distinguish three patterns of architectures with increasing genomic background mutation rate Θ_*bg*_: complete sweeps, for Θ_*bg*_ ≲ 0.1, heterogeneous partial sweeps at several loci for 0.1 < Θ_*bg*_ < 100, and polygenic frequency shifts for Θ_*bg*_ ≳ 100. The plots show the marginal distributions of all loci, ordered according to their allele frequency, i.e. the major locus in red and all following (first, second, third, etc. minors) in blue to green to yellow. Lines in respective colors show analytical predictions, see S1 Appendix, Section E. Simulations were stopped once the populations have adapted to 95% of the maximum mean fitness in each of 10 000 replicates, resulting in an the upper bound for the major locus distribution at, *p*_1_ = 0.95. Simulations for *s*_*b*_ = −*s*_*d*_ = 0.1. Note the different scaling of the y-axis (density, normalized to 1 per locus) for different mutation rates.

Fig 4 displays the main result of this section. It shows the marginal distributions of all loci, ordered according to their allele frequency at the time of observation (major locus, 1st, 2nd, 3rd minor locus, *etc*.) for traits with *L* = 2, 10, 50, and 100 loci. Panels in the same row correspond to equal background mutation rates Θ_*bg*_ = (*L* − 1)Θ_*l*_, but note that the locus mutation rates Θ_*l*_ are not equal. The figure reveals a striking level of uniformity of adaptive architectures with the same Θ_*bg*_, but vastly different number of loci. For Θ_*bg*_ ≤ 1 (the first three rows), the marginal distributions for loci of the same order (same color in the Figure) across traits with different *L* is almost invariant. For large Θ_*bg*_, they converge for sufficiently large *L* (e.g. for Θ_*bg*_ = 10, going from *L* = 10 to *L* = 50 and to *L* = 100). In particular, the background mutation rate Θ_*bg*_ determines the shape of the major-locus distribution (red in the Figure) for high *p* → 1 − *f*_*w*_ = 0.95 (the maximum possible frequency, given the stopping condition). For Θ_*bg*_ < 1, this distribution is sharply peaked with a singularity at *p* = 1 − *f*_*w*_, whereas it drops to zero for high *p* if Θ_*bg*_ > 1 (see also the analytical results below).

As predicted by the theory, Eq (8) and below, simulations confirm that the overall selection strength does not affect the adaptive architecture (see S1 Fig for comparison of simulation results for *s*_*b*_ = 0.1 and *s*_*b*_ = 0.01). As discussed in S1 Appendix, Section A, sufficiently tight linkage does change the shape of the distributions. Importantly, however, it does not affect the role of Θ_*bg*_ in determining the singularity of the major-locus distribution. This confirms the key role of the background mutation rate as a single parameter to determine the adaptive scenario in our model. While Θ_*bg*_ = 1 separates architectures that are dominated by a single major locus (Θ_*bg*_ < 1) from collective scenarios (with Θ_*bg*_ > 1), the classical sweep or shift scenarios are only obtained if Θ_*bg*_ deviates strongly from 1. We therefore distinguish three adaptive scenarios.

- Θ_*bg*_ ≲ 0.1, *single completed sweeps*. For Θ_*bg*_ ≪ 1 (first two rows of Fig 4), the distribution of the major locus is concentrated at the maximum of its range, while all other distributions are concentrated around 0. Adaptation thus occurs at a single locus, via a selective sweep from low to high mutant frequency. Contributions by further loci are rare. If they occur at all they are usually due to a single runner-up locus (the highest minor locus).
- 0.1 < Θ_*bg*_ < 100, *heterogeneous partial sweeps*. With intermediate background mutation rates (third and forth row of Fig 4), we still observe a strong asymmetry in the frequency spectrum. Even for Θ_*bg*_ = 10, there is a clear major locus discernible, with most of its distribution for *p* > 0.5 However, there is also a significant contribution of several minor loci that rise to intermediate frequencies. We thus obtain a heterogeneous pattern of partial sweeps at a limited number of loci.
- Θ_*bg*_ ≳ 100, *homogeneous frequency shifts*. Only for high background mutations rates Θ_*bg*_ ≫ 1 (last row of Fig 4 with Θ_*bg*_ = 100), the heterogeneity in the locus contributions to the adaptive response vanishes. There is then no dominating major locus. For only 2 loci, these shifts are necessarily still quite large, but for traits with a large genetic basis (large *L*; the only realistic case for high values of Θ_*bg*_), adaptation occurs via subtle frequency shifts at many loci.

#### Analytical predictions

To gain deeper understanding of the polygenic architecture—and for quantitative predictions—we dissect our analytical result for the joint frequency spectrum in Eq (8). We start with the case of *L* = 2 loci, allowing for different locus mutation rates Θ_1_ and Θ_2_. The marginal distribution at the first locus reads (from Eq (8), after integration over *p*_2_),

$$\begin{array}{c} {\text{P}}_{\;f_{w}}\left[p_{1}|\Theta_{1},\Theta_{2}\right]=\frac{p_{1}^{\Theta_{1}-1}{\left(1-p_{1}-f_{w}\right)}^{\Theta_{2}-1}{\left(1-p_{1}\right)}^{\Theta_{1}+1}}{B\left[\Theta_{1},\Theta_{2}\right]\;{\left(1-p_{1}^{2}-f_{w}\right)}^{\Theta_{1}+\Theta_{2}}}(1-\frac{f_{w}\left(1-2p_{1}\right)}{{\left(1-p_{1}\right)}^{2}}), \end{array}$$ (11)

for 0 ≤ *p*_1_ ≤ 1 − *f*_*w*_ (see also S1 Appendix, Section F). The distribution has a singularity at *p*_1_ = 0 if the corresponding *locus* mutation rate is smaller than one, Θ_1_ < 1. It has a singularity at *p*_1_ = 1 − *f*_*w*_ if the corresponding *background* mutation rate (which is just the mutation rate at the other locus for *L* = 2) is smaller than one, Θ_2_ < 1. The marginal distributions at the major locus, ${\text{P}}_{\;f_{w}}^{+}\left[p|\Theta_{1},\Theta_{2}\right]$, and the minor locus, ${\text{P}}_{\;f_{w}}^{-}\left[p|\Theta_{1},\Theta_{2}\right]$, follow from Eq (11) as

$$\begin{array}{c} {\text{P}}_{\;f_{w}}^{\pm }\left[p|\Theta_{1},\Theta_{2}\right]={\text{P}}_{\;f_{w}}\left[p|\Theta_{1},\Theta_{2}\right]+{\text{P}}_{\;f_{w}}\left[p|\Theta_{2},\Theta_{1}\right], \end{array}$$ (12)

where ${\text{P}}_{\;f_{w}}^{+}\left[p|\Theta_{1},\Theta_{2}\right]$ is defined for $1-\sqrt{f_{w}}\leq p\leq 1-f_{w}$ and ${\text{P}}_{\;f_{w}}^{-}\left[p|\Theta_{1},\Theta_{2}\right]$ is defined for $0\leq p\leq 1-\sqrt{f_{w}}$. The sum in Eq (12) accounts for the alternative events that either the first or the second locus may end up as the major (or minor) locus. Consequently, ${\text{P}}_{\;f_{w}}^{-}\left[p|\Theta_{1},\Theta_{2}\right]$ has a singularity at *p* = 0 if the *minimal locus mutation rate* Θ_*l*_ = min[Θ_1_, Θ_2_] < 1. Analogously, ${\text{P}}_{\;f_{w}}^{+}\left[p|\Theta_{1},\Theta_{2}\right]$ has a singularity at *p* = 1 − *f*_*w*_ if the *minimal background mutation rate* Θ_*bg*_ = min[Θ_1_, Θ_2_] < 1. The left column of Fig 4 shows the distributions at the major and minor locus for *L* = 2 in the symmetric case Θ_1_ = Θ_2_ = Θ_*l*_ = Θ_*bg*_ and *f*_*w*_ = 0.05. Simulations for a population of size *N*_*e*_ = 10 000 and analytical predictions match well.

How do these results generalize for *L* > 2? We again allow for unequal locus mutation rates Θ_*i*_. It is easy to see from Eq (8) that the marginal distribution at the *i*th locus has a singularity at *p*_*i*_ = 0 for Θ_*i*_ < 1. In S2 Appendix, Section M.3, we further show that it has a second singularity at *p*_*i*_ = 1 − *f*_*w*_ if the corresponding background mutation rate $\sum_{j\neq i}^{d}\Theta_{j}$ is smaller than 1. As a first step, we split the joint distribution, Eq (8), into the marginal distribution at the major locus ${\text{P}}_{\;f_{w}}^{+}\left[p|\Theta \right]$ (defined for $1-\sqrt[{L}]{f_{w}}\leq p\leq 1-f_{w}$) and a cumulative distribution at all other (minor) loci, ${\text{P}}_{\;f_{w}}^{-}\left[p|\Theta \right]$ (defined for $0\leq p\leq 1-\sqrt{f_{w}}$). Since any locus can end up as the major locus (with probability > 0), ${\text{P}}_{\;f_{w}}^{+}\left[p|\Theta \right]$ has a singularity at *p* = 1 − *f*_*w*_ for

$$\begin{array}{c} \Theta_{bg}≔\underset{1\leq i\leq L}{\text{min}}[\sum_{j=1}^{L}\Theta_{j}-\Theta_{i}]<1\;. \end{array}$$ (13)

This equation generalizes the definition of the background mutation rate, Eq (10), to the case of unequal locus mutation rates. Similarly, ${\text{P}}_{\;f_{w}}^{-}\left[p|\Theta \right]$ has a singularity at *p* = 0 if

$$\begin{array}{c} \Theta_{l}≔\underset{1\leq i\leq L}{\text{min}}[\Theta_{i}]<1\;. \end{array}$$ (14)

As long as Θ_*bg*_ ≤ 1, we can approximate both the major-locus distribution ${\text{P}}_{\;f_{w}}^{+}\left[p|\Theta \right]$ and the cumulative minor locus distribution ${\text{P}}_{\;f_{w}}^{-}\left[p|\Theta \right]$ for arbitrary *L* by formulas for a 2-locus model with locus mutation rates matching Θ_*l*_ and Θ_*bg*_ of the multi-locus model, Eq (12). Similarly, we can use results from a *k*-locus model to match the marginal distributions of the largest *k* loci (i.e., up to the (*k* − 1)th minor) in models with *L* > *k* loci, upon rescaling of the mutation rates. As explained for the ratio of the first minor and major locus in the previous section, rescaling rules match the expected waiting time for a mutation (destined for establishment) at the *k*th locus after the origin of a first mutation. Details are given in the S1 Appendix, Section E. In Fig 4, we use formulas derived from a *k*-locus model (*k* ≤ 4) to approximate the (*k* − 1)st minor locus distribution of models with *L* = 10; 50; 100 loci and Θ_*bg*_ ≤ 1. These approximations work well as long as these leading loci dominate the adaptive architecture of the trait, which is the case for Θ_*bg*_ ≤ 1.

### Relaxing complete redundancy

To complete our picture of adaptive architectures, we investigate the robustness of our model assumption against relaxation of redundancy. As explained above (*Model extensions* and Fig 1), we implement diminishing returns epistasis, such that an individual with a single mutation has fitness *δs*_*b*/*d*_, while individuals carrying more than one mutation have fitness *s*_*b*/*d*_. With small deviations from complete redundancy (e.g. *δ* = 0.9, stopping at 5% ancestral phenotypes, see Fig S2 Fig) we obtain basically no differences in the genomic patterns of adaptation. With larger deviations (e.g. *δ* = 0.5) quantitative differences appear. However, the qualitative picture concerning the scenario of polygenic adaptation remains the same.

**Fig 5:**
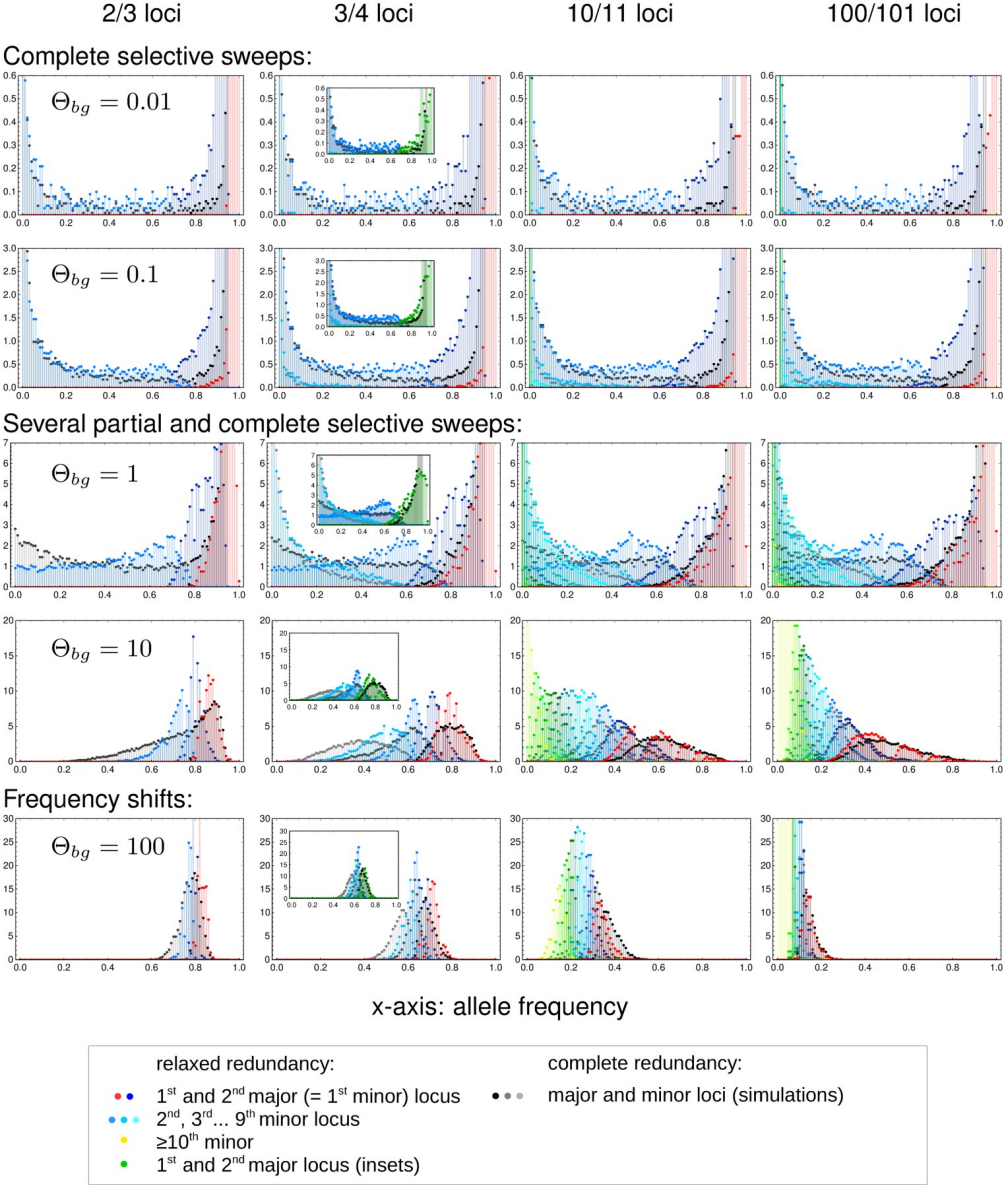
Relaxed redundancy. Relaxing redundancy such that a single mutant has fitness 1 + 0.5*s*_*b*/*d*_ and only two mutations or more confer the full fitness effect (1 + *s*_*b*/*d*_) demonstrates the robustness of our model. As in Fig 4, allele frequency distributions of derived alleles are displayed once the population has reached 95% of maximum attainable mean population fitness. Genomic patterns of adaptation show similar characteristics as with complete redundancy. Due to relaxed redundancy, an additional “major locus” is required to reach the adaptive optimum. As explained in the main text, the distribution of the *k*th largest locus with complete redundancy therefore corresponds to the distribution of the *k* + 1st largest locus with relaxed redundancy. Insets in the second column show the same data with the distributions of the two major loci for relaxed redundancy combined (in green).

Fig 5 shows the marginal frequency distributions of major and minor loci for a trait with relaxed redundancy with *δ* = 0.5 that is sampled when the population has accomplished 95% of the fitness increase on its way to the new optimum, Eq (2). Given the fitness function, this is not possible with adaptation at only a single locus. At least two loci are needed. The Figure compares the simulation data for the relaxed redundancy model (colored dots) and the full redundancy model (dots in back and gray). As in Fig 4, traits in the same row have the same background mutation rate Θ_*bg*_. However, the background rate for the model with relaxed redundancy is redefined as

$$\begin{array}{c} \Theta_{bg}^{\text{relax}}=\left(L-2\right)\Theta_{l}, \end{array}$$ (15)

where Θ_*l*_ is the locus mutation rate (equal at all loci). We thus define the background rate, more precisely, as the combined population-scaled mutation rate of all loci *that are not essential* to accomplish adaptation of the phenotype and, thus, are truly redundant. With this choice, the adaptive architecture of the relaxed redundancy model reproduces the one of the model with full redundancy—up to a shift in the number of the loci due to an extra locus that is needed for adaptation with relaxed redundancy. The Figure captures this by comparing traits with relaxed redundancy with *L* = 3, 4, 11, and 101 loci to fully redundant traits with one fewer locus. The inset figures in the column for *L* = 4 loci show the same scenario, but with an *averaged* marginal distribution for the two largest loci with relaxed redundancy (in green).

- For mutation rates, Θ_*bg*_ ≪ 1, we still find adaptation by sweeps. Relative to the full redundancy model, we now observe two “major” sweep loci instead of only a single sweep. The inset (for *L* = 4) shows that their averaged distributions matches the major locus distribution of the full redundancy model. The distribution at the third largest locus (the “first minor” locus with relaxed redundancy) resembles the corresponding distribution of the first minor locus of the trait with full redundancy.
- For intermediate mutation rates, 0.1 < Θ_*bg*_ < 100, the pattern is dominated by partial sweeps. We clearly see the similarity in the marginal distributions of the *k*th largest locus with full redundancy and the *k* + 1st largest locus of the relaxed redundancy trait. For the two major loci with relaxed redundancy, we again see (inset) that the averaged distribution matches the major-locus distribution of the full redundancy model.
- Finally, for strong mutation, Θ_*bg*_ ≳ 100, adaptation again occurs by small frequency shifts at many loci.

In summary, our results show that relaxing redundancy leads to qualitatively similar results, but with a reduced “effective” background mutation rate that only accounts for “truly redundant” loci.

## Discussion

Traits with a polygenic basis can adapt in different ways. Few or many loci can contribute to the adaptive response. The changes in the allele frequencies at these loci can be large or small. They can be homogeneous or heterogeneous. While molecular population genetics posits large frequency changes—selective sweeps—at few loci, quantitative genetics views polygenic adaptation as a collective response, with small, homogeneous allele frequency shifts at many loci. Here, we have explored the conditions under which each adaptive scenario should be expected, analyzing a polygenic trait with redundancy among loci that allows for a full range of adaptive architectures: from sweeps to subtle frequency shifts.

### Polygenic architectures of adaptation

For any polygenic trait, the multitude of possible adaptive architectures is fully captured by the joint distribution of mutant alleles across the loci in its basis. Different adaptive scenarios (such as sweeps or shifts) correspond to characteristic differences in the shape of this distribution, at the end of the adaptive phase. For a single locus, the stationary distribution under mutation, selection, and drift can be derived from diffusion theory and has been known since the early days of population genetics (S. Wright (1931), [32]). For multiple interacting loci, however, this is usually not possible. To address this problem for our model, we dissect the adaptive process into two phases. The early stochastic phase describes the establishment of all mutants that contribute to the adaptive response under the influence of mutation and drift. We use that loci can be treated as independent during this phase to derive a joint distribution for ratios of allele frequencies at different loci, Eq (5). During the second, deterministic phase, epistasis and linkage become noticeable, but mutation and drift can be ignored. Allele frequency changes during this phase can be described as a density transformation of the joint distribution. For the simple model with fully redundant loci, and assuming either LE or complete linkage, this transformation can be worked out explicitly. Our main result Eq (8) can be understood as a multi-locus extension of Wright’s formula. For a neutral locus with multiple alleles, Wright’s distribution is a Dirichlet distribution, which is reproduced in our model for the case of complete linkage, see S1 Appendix, Section A. For the opposite case of linkage equilibrium, we obtain a family of inverted Dirichlet distributions, depending on the stopping condition—our time of observation.

Note that (unlike Wright’s distribution) the distribution of adaptive architectures is *not a stationary distribution, but necessarily transient*. It describes the pattern of mutant alleles at the end of the “rapid adaptive phase” [30, 31], because this is the time scale that the opposite narratives of population genetics and quantitative genetics refer to. In particular, the quantitative genetic “small shifts” view of adaptation does not talk about a stationary distribution: it does not imply that alleles will never fix over much longer time scales, due to drift and weak selection. On a technical level, the transient nature of our result means that it reflects the effects of genetic drift only during the early phase of adaptation. These early effects are crucial because they are magnified by the action of positive selection. In contrast, our result ignores drift after phenotypic adaptation has been accomplished—which is also a reason why it can be derived at all.

To capture the key characteristics of the adaptive architecture, we dissect the joint distribution in Eq (8) into marginal distributions of single loci. As explained at the start of the results section, these loci do not refer to a fixed genome position, but are defined *a posteriori* via their role in the adaptive process. For example, the *major locus* is defined as the locus with the highest mutant allele frequency at the end of the adaptive phase. (Since all loci have equal effects in our model, this is also the locus with the largest contribution to the adaptive response, but see S1 Appendix, Section D.) This is a different way to summarize the joint distribution than used in some of the previous literature [26, 28, 29], which rely on a gene-centered view to study the pattern at a focal locus, irrespective of its role in trait adaptation. In contrast, we use a trait-centered view, which is better suited to describe and distinguish adaptive scenarios. For example, “adaptation by sweeps” refers to a scenario where sweeps happen at some loci, rather than at a specific locus. This point is further discussed in S1 Appendix, Section F, where we also display marginal distributions of Eq (8) for fixed loci.

#### The role of the background mutation rate

Our results show that the qualitative pattern of polygenic adaptation is predicted by a single compound parameter: the background mutation rate Θ_*bg*_ (see Eqs (10), (13) and (15)), i.e., the population mutation rate for the background of a focal locus within the trait basis. For a large basis, Θ_*bg*_ is closely related to the trait mutation rate. We can understand the key role of this parameter as follows. As detailed in the Section *Analytical analysis*, the early stochastic phase of adaptation is governed by two processes: New successful mutations (destined for establishment) enter the population at rate Θ_*l*_*s*_*b*_ per locus (where Θ_*l*_ is the locus mutation rate and *s*_*b*_ the selection coefficient), while existing mutants spread with an exponential rate *s*_*b*_. Consider the locus that carries the first successful mutant. For Θ_*bg*_ < 1, the expected spread from this first mutant exceeds the creation of new mutant lineages at all other loci. Therefore, the locus will likely maintain its lead, with an exponentially growing gap to the second largest locus. Vice versa, for Θ_*bg*_ > 1, most likely one of the competing loci will catch up. We can thus think of Θ_*bg*_ as a measure of competition experienced by the major locus due to adaptation at redundant loci in its genetic background. The argument does not depend on the strength of selection, which affects both rates in the same way. The same can be shown for adaptation from standing genetic variation at mutation-selection-drift balance, see S2 Appendix, Section M.1. As a consequence of low mutant frequencies during the stochastic phase, the result is also independent of interaction effects due to epistasis or linkage.

Since the order of loci is not affected by the deterministic phase of the adaptive process, Θ_*bg*_ maintains its key role for the adaptive architecture. In the joint frequency distribution, Eqs (5) and (8), it governs the singular behavior of the marginal distribution at the major locus. For Θ_*bg*_ < 1, this distribution has a singularity at the maximum of its range. Adaptation is therefore dominated by the major locus, leading to heterogeneous architectures. For Θ_*bg*_ ≲ 0.1, adaptation occurs almost always due to a completed sweep at this locus. For Θ_*bg*_ > 1, in contrast, no single dominating locus exists: adaptation is collective and supported by multiple loci. For a polygenic trait with Θ_*bg*_ ≳ 100, we obtain homogeneous small shifts at many loci, as predicted by quantitative genetics.

The result also shows that the adaptive scenario does not depend directly on the number of loci in the genetic basis of the trait, but rather on their combined mutation rate (the mutational target size, *sensu* [11]). For redundant loci and fixed Θ_*bg*_, the predicted architecture at the loci with the largest contribution to the adaptive response is almost independent of the number of loci, see Fig 4. Qualitatively, the same still holds true when the assumption of complete redundancy is dropped (Fig 5). In this case, only loci in the genetic background that are not required to reach the new trait optimum, but offer redundant routes for adaptation, are included in Θ_*bg*_. Note that the same reasoning holds for a quantitative trait that is composed of several modules of mutually redundant genes, but where interactions among genes in different modules only affect a focal module as a unit. I.e., due to changes in the genetic background, all loci in this module experience a uniform change in the selection coefficient *s*_*b*_ = *s*_*b*_(*t*) > 0. In this case, assuming LE, our model still applies (cf. S2 Appendix). The adaptive architecture for each module depends only on the module-specific Θ_*bg*_, but not on the mutation rates at genes in the basis of the trait outside of the module. Finally, we note that related measures of genetic redundancy have previously been shown to determine the genetic architecture of local adaptation in the face of gene flow [40].

#### Polygenic adaptation and soft sweeps

In our analysis of polygenic adaptation, we have not studied the probability that adaptation at single loci could involve more than a single mutational origin and thus produces a so-called *soft selective sweep from recurrent mutation*. As explained in [6, 41], however, the answer is simple and only depends on the locus mutation rate—independently of adaptation at other loci. Soft sweeps become relevant for Θ_*l*_ ≳ 0.1. For much larger values Θ_*l*_ ≫ 1, they become “super-soft” in the sense that single sweep haplotypes do not reach high frequencies because there are so many independent origins of the mutant allele. The role of Θ_*bg*_ for polygenic adaptation is essentially parallel to the one of Θ_*l*_ for soft sweeps. In both cases, the population mutation rate is the only relevant parameter, with a lower threshold of Θ ∼ 0.1 for a signal involving multiple alleles and much higher values for a “super-soft” scenario with only subtle frequency shifts. Nevertheless, the mathematical methods to analyze both cases are different, essentially because the polygenic scenario does not lend itself to a coalescent approach.

### Alternative approaches to polygenic adaptation

The theme of “competition of a single locus with its background” relates to previous findings by Chevin and Hospital (2008) [26] in one of the first studies to address polygenic footprints. These authors rely on a deterministic model of an additive quantitative trait to describe the adaptive trajectory at a single target QTL in the presence of background variation. The background is modeled as a normal distribution with a mean that can respond to selection, but with constant variance. Obviously, a drift-related parameter, such as Θ_*bg*_, has no place in such a framework. Still, there are several correspondences to our result on a qualitative level. Specifically, a sweep at the focal locus is prohibited under two conditions. First, the background variation (generated by recurrent mutation in our model, constant in [26]) must be large. Second, the fitness function must exhibit strong negative epistasis that allows for alternative ways to reach the trait optimum—and thus produces redundancy (due to Gaussian stabilizing selection in [26]). Finally, while the adaptive trajectory depends on the *shape* of the fitness function, Chevin and Hospital note that it does not depend on the *strength* of selection on the trait, as also found for our model.

A major difference of the approach used in [26] is the gene-centered view that is applied there. Consider a scenario where the genetic background “wins” against the focal QTL and precludes it from sweeping. For a generic polygenic trait (and for our model) this still leaves the possibility of a sweep at one of the background loci. However, this is not possible in [26], where all background loci are summarized as a sea of small-effect loci with constant genetic variance.

This constraint is avoided in the approach by deVladar and Barton [42] and Jain and Stephan [31], who study an additive quantitative trait under stabilizing selection with binary loci (see also [43] for an extension to adaptation to a moving optimum). These models allow for different locus effects, but ignore genetic drift. Before the environmental change, all allele frequencies are assumed to be in mutation-selection balance, with equilibrium values derived in [42]. At the environmental change, the trait optimum jumps to a new value and alleles at all loci respond by large or small changes in the allele frequencies. Overall, [42] and [31] predict adaptation by small frequency shifts in larger parts of the biological parameter space relative to our model. In particular, sweeps are prevented in [31] if most loci have a small effect and are therefore under weak selection prior to the environmental change. This contrasts to our model, where the predicted architecture of adaptation is independent of the selection strength. Thus, in our model, weak selection does not imply shifts. This difference can at least partially be explained by the neglect of drift effects on the starting allele frequencies in the deterministic models. In the absence of drift, loci under weak selection start out from frequency *x*_0_ = 0.5 [42]. In finite populations, however, almost all of these alleles start from very low (or very high) frequencies—unless the population mutation parameter is large (many alleles at intermediate frequencies at competing background loci are expected only if Θ_*bg*_ ≫ 1, in accordance with our criterion for *shifts*). To test this further, we have analyzed our model for the case of starting allele frequencies set to the deterministic values of mutation-selection balance, *μ*/*s*_*d*_. Indeed, we observe adaptation due to small frequency shifts in a much larger parameter range (S1 Appendix, Section B).

Generally, adaptation by sweeps in a polygenic model requires a mechanism to create heterogeneity among loci. This mechanism is entirely different in both modeling frameworks. While heterogeneity is (only) produced by unequal locus effects for the deterministic quantitative trait, it is (solely) due to genetic drift for the redundant trait model. Since both approaches ignore one of these factors, both results should rather underestimate the prevalence of sweeps. Indeed, heterogeneity increases for our model with unequal locus effects (see S1 Appendix, Section D).

Both drift and unequal locus effects are included in the simulation studies by Pavlidis et al (2012) [28] and Wollstein and Stephan (2014) [29]. These authors assess patterns of adaptation for a quantitative trait under stabilizing selection with up to eight diploid loci. However, due to differences in concepts and definitions there are few comparable results. In contrast to [31] and to our approach, they study long-term adaptation (they simulate *N*_*e*_ generations). In [28, 29], *sweeps* are defined as fixation of the mutant allele at a focal locus, whereas *frequency shifts* correspond to long-term stable polymorphic equilibria [29]. With this definition, a *shift* scenario is no longer a transient pattern, but depends entirely on the existence (and range of attraction) of polymorphic equilibria. A polymorphic outcome is likely for a two-locus model with full symmetry, where the double heterozygote has the highest fitness. For more than two loci, the probability of shifts *decreases* (because polymorphic equilibria become less likely, see [44]). However, also the probability of a sweep decreases. This is largely due to the gene-centered view in [28], where potential sweeps at background loci are not recorded (see also S1 Appendix, Section F).

### Scope of the model and the analytical approach

We have described scenarios of adaptation for a simple model of a polygenic trait. This model allows for an arbitrary number of loci with variable mutation rates, haploids and diploids, linkage, time-dependent selection, new mutations and standing genetic variation, and alternative starting conditions for the mutant alleles. Its genetic architecture, however, is strongly restricted by our assumption of (full or relaxed) redundancy among loci. In the haploid, fully redundant version, the phenotype is binary and only allows for two states, *ancestral wildtype* and *mutant*. Biologically, this may be thought of as a simple model for traits like pathogen or antibiotic resistance, body color, or the ability to use a certain substrate [45, 46].

Our main motivation, however, has been to construct a minimal model with a polygenic architecture that allows for both sweep and shifts scenarios—and for comprehensive analytical treatment. One may wonder how our methods and results generalize if we move beyond our model assumptions.

Key to our analytical method is the dissection of the adaptive process into a stochastic phase that explains the origin and establishment of beneficial variants and a deterministic phase that describes the allele frequency changes of the established mutant copies. This framework can be applied to a much broader class of models. Indeed, in many cases, the fate of beneficial alleles, establishment or loss, is decided while these alleles are rare. Excluding complex scenarios such as passage through a fitness valley, the initial stochastic phase is relatively insensitive to interactions via epistasis or linkage. We can therefore describe the dynamics of traits with a different architecture (e.g. an additive quantitative trait with equal-effect loci under stabilizing selection) within the same framework by coupling the same stochastic dynamics to a different set of differential equations describing the dynamics during the deterministic phase.

This is important because, as described above, the key *qualitative* results to distinguish broad categories of adaptive scenarios are due to the initial stochastic phase. This holds true, in particular, for the role of the background mutation rate Θ_*bg*_. We therefore expect that these results generalize beyond our basic model. Indeed, we have already seen this for our model extensions to include diploids, linkage, and relaxed redundancy. Vice-versa, we have seen that other factors, such as alternative starting conditions for the mutant alleles, directly affect the early stochastic phase and lead to larger changes in the results. As shown in S1 Appendix, Section B, however, they can be captured by an appropriate extension of the stochastic Yule process framework.

Several factors of biological importance are not covered by our current approach. Most importantly, this includes loci with different effect sizes and spatial population structure. Both require a further extension of our framework for the early stochastic phase of adaptation. Unequal locus effects (both directly on the trait or on fitness due to pleiotropy) are expected to enhance the heterogeneity in the adaptive response among loci, as confirmed by simulations of a 2-locus model in S1 Appendix, Section D. The opposite is true for spatial structure, as further discussed below.

### When to expect sweeps or shifts

Although our assumptions on the genetic architecture of the trait (complete redundancy and equal loci) are favorable for a collective, shift-type adaptation scenario, we observe large changes in mutant allele frequencies (completed or partial sweeps) for major parts of the parameter range. A homogeneous pattern of *subtle frequency shifts* at many loci is only observed for high mutation rates. This contrasts with experience gained from breeding and modern findings from genome-wide association studies, which are strongly suggestive of an important role for small shifts with contributions from very many loci (reviewed in [1, 15, 47–49], see [12, 50, 51] for recent empirical examples). For traits such as human height, there has even been a case made for *omnigenic* adaptation [8], setting up a “mechanistic narrative” for Fisher’s (conceptual) infinitesimal model. Clearly, body height may be an extreme case and the adaptive scenario will strongly depend on the type of trait under consideration. Still, the question arises whether and how wide-spread shift-type adaptation can be reconciled with our predictions. We will first discuss this question within the scope of our model and then turn to factors beyond our model assumptions.

#### The size of the background mutation rate

The decisive parameter to predict the adaptive scenario in our model, the background mutation rate, is not easily amenable to measurement. Θ_bg_ = (*L* − 1)Θ_*l*_ compounds two factors, the locus mutation parameter Θ_*l*_ and the number of loci *L*, which are both complex themselves and require interpretation. To assess the plausibility of values of the order of Θ_*bg*_ ≳ 100, required for homogeneous polygenic shifts in our model, we consider both factors separately.

Large locus mutation rates Θ_*l*_ = 4*N*_*e*_*μ* (for diploids, 2*N*_*e*_*μ* for haploids) are possible if either the allelic mutation rate *μ* or the effective population size *N*_*e*_ is large. Both cases are discussed in detail (for the case of soft sweeps) in [6]. Basically, *μ* can be large if the mutational target *at the locus* is large. Examples are loss-of-function mutations or cis-regulatory mutations. *N*_*e*_ is the *short-term effective population size* [41, 52, 53] during the stochastic phase of adaptation. This *short-term* size is unaffected by demographic events, such as bottlenecks, prior to adaptation. It is therefore often larger than the *long-term* effective size that is estimated from nucleotide diversity. (Strong changes in population size *during* the adaptive period can have more subtle effects [54].) For recent adaptations due to gain-of-function mutations, plausible values are Θ_*l*_ ≲ 0.1 for *Drosophila* and Θ_*l*_ ≲ 0.01 for humans [6].

If 10 000 loci or more contribute to the basis of a polygenic trait [8], large values of Θ_*bg*_ could, in principle, easily be obtained. However, the parameter *L* in our model counts only loci that actually can respond to the selection pressure: mutant alleles must change the trait in the right direction and should not be constrained by pleiotropic effects. Omnigenic genetics, in particular, also implies ubiquitous pleiotropy and so the size of the basis *that is potentially available for adaptation* is probably strongly restricted. For a given trait, the number of available loci *L* may well differ, depending on the selection pressure and pleiotropic constraints. Furthermore, our results for the model with relaxed redundancy show that Θ_*bg*_ only accounts for loci that are truly redundant and offer alternative routes to the optimal phenotype. With this in mind, values of *L* in the hundreds or thousands (required for Θ_*bg*_ ≥ 100) seem to be quite large. While some highly polygenic traits such as body size could still fulfill this condition, this appears questionable for the generic case.

#### Balancing selection and spatial structure

In our model, characteristic patterns in the adaptive architecture result from heterogeneities among loci that are created by mutation and drift during the initial stochastic phase of adaptation. As initial condition, we have mostly assumed that mutant alleles segregate in the population in the balance of mutation, purifying selection and genetic drift. Since this typically results in a broad allele frequency distribution (unless mutation is very strong), it favors heterogeneity among loci and thus adaptation by (partial) sweeps. However, even after decades of research, the mechanisms to maintain genetic variation in natural populations remain elusive [1]. As discussed in S1 Appendix, Section B, more homogeneous starting conditions for the mutant alleles can be strongly favorable of a shift scenario. Such conditions can be created either by balancing selection or by spatial population structure.

Balancing selection (due to overdominance or negative frequency dependence) typically maintains genetic variation at intermediate frequencies. If a major part of the genetic variance for the trait is due to balancing selection, adaptation could naturally occur by small shifts. However, the flexibility of alleles at single loci, and thus the potential for smaller or larger shifts, will depend on the strength of the fitness trade-off (e.g. due to pleiotropy) at each locus. If these trade-offs are heterogeneous, the adaptive architecture will reflect this. Also, adaptation against a trade-off necessarily involves a fitness cost. Therefore, if the trait can also adapt at loci that are free of a trade-off, these will be preferred, possibly leading to sweeps.

As discussed in a series of papers by Ralph and Coop [34, 35], spatial population structure is a potent force to increase the number of alternative alleles that contribute to the adaptive response. If adaptation proceeds independently, but in parallel, in spatially separated subpopulations, different alleles may be picked up in different regions. Depending on details of the migration pattern [36], we then expect architectures that are globally polygenic with small shifts, but locally still show sweeps or dominating variants.

Furthermore, population structure and gene flow *before* the start of the selective phase can have a strong effect on the starting frequencies. In particular, if the base population is admixed, mutant alleles could often start from intermediate frequencies and naturally produce small shifts. This applies, in particular, to adaptation in modern human populations, which have experienced major admixture events in their history [55, 56] and only show few clear signals of selective sweeps [11].

Finally, gene flow and drift will continue to change the architecture of adaptation after the rapid adaptive phase that has been our focus here. This can work in both directions. On the one hand, subsequent gene flow can erase any *local* sweep signals by mixing variants that have been picked up in different regions [34, 35]. On the other hand, local adaptation, in particular, may favor adaptation by large-effect alleles at few loci, favoring sweeps over longer time-scales. Indeed, as argued by Yeaman [40], initial rapid adaptation due to small shifts at many alleles of mostly small effect may be followed by a phase of allelic turnover, during which alleles with small effect are swamped and few large-effect alleles eventually take over. This type of allele sorting over longer time-scales is also observed in simulations studies for a quantitative trait under stabilizing selection that adapts to a new optimum after an environmental change [31, 57].

#### Between sweeps and shifts: Adaptation by partial sweeps

Previous research has almost entirely focused on either of the two extreme scenarios for adaptation: sweeps in a single-locus setting or (infinitesimal) shifts in the tradition of Fisher’s infinitesimal model. This leaves considerable room for intermediate patterns. Our results for the redundant trait model show that such transitional patterns should be expected in a large and biologically relevant parameter range (values of Θ_*bg*_ between 0.1 and 100). Patterns between sweeps and shifts are *polygenic* in the sense that they result from the *concerted* change in the allele frequency at multiple loci. They can only be understood in the context of interactions among these loci. However, they usually do not show subtle shifts, but much larger changes (partial sweeps) at several loci. If adaptation occurs from mutation-selection-drift balance, the polygenic patterns are typically strongly heterogeneous, even across loci with identical effects on the trait. Such patterns may be difficult to detect with classical sweep scans, in particular if partial sweeps are “soft” because they originate from standing genetic variation or involve multiple mutational origins. However, they should be visible in time-series data and may also leave detectable signals in local haplotype blocks.

Indeed there is empirical evidence for partial sweeps from time series data in experimental *evolve and resequence* experiments on recombining species such as fruit flies. For example, Burke *et al*. [58] observe predominantly partial sweeps (from SGV) in their long-term selection experiments with *Drosophila melanogaster* for accelerated development—a rather unspecific trait with a presumably large genomic basis. A similar pattern of “plateauing”, where allele frequencies at several loci increase quickly over several generations, but then stop at intermediate levels, was recently observed by Barghi and collaborators [59] for adaptation of 10 *Drosophila simulans* replicates to a hot temperature environment. Complementing the genotypic time-series data with measurements of several phenotypes, these authors found convergent evolution for several high-level traits (such as fecundity and metabolic rate), indicating that rapid phenotypic adaptation had reached a new optimum. This high-level convergence contrasts a strong heterogeneity in the adaptation response among loci and also between replicates [59]. Based on their data, the authors reject both a selective sweep model and adaptation by subtle shifts. Instead, the observed patterns are most consistent with the intermediate adaptive scenario in our framework, featuring heterogeneous partial sweeps at interacting loci with a high level of genetic redundancy.

## Supporting information

S1 Appendix Appendix. Model extensions and supporting analysis. Extensions of the model to include (A) linkage, (B) alternative starting frequencies, (C) diploids, and (D) asymmetric loci. Detailed analysis of (E) approximations for multi-locus architectures, (F) the marginal distribution of a single locus, and (G) the dynamics of adaptation.
(PDF)

S2 Appendix Mathematical Appendix. Detailed description and derivation of the analytical results.
(PDF)

S1 Fig Architecture of polygenic adaptation for weaker selection. (PDF)

S2 Fig Architecture with weakly relaxed redundancy. (PDF)

S1 File Mathematica notebook. *Mathematica* [60] notebook to produce the analytical approximations in all figures.
(NB) Click here for additional data file.
