# Supplementary material for: Polygenic adaptation: From sweeps to subtle frequency shifts
Source: PLoS Genet. 2019 Mar 20;15(3):e1008035. doi: 10.1371/journal.pgen.1008035 (PMC6443195; doi:10.1371/journal.pgen.1008035)
Supplement: S1 Appendix — Extensions of the model to include (A) linkage, (B) alternative starting frequencies, (C) diploids, and (D) asymmetric loci. Detailed analysis of (E) approximations for multi-locus architectures, (F) the marginal distribution of a single locus, and (G) the dynamics of adaptation. (PDF) [file pgen.1008035.s001.pdf]

S1 Appendix for *Polygenic adaptation: From sweeps to subtle frequency shifts*

Ilse Höllinger, Pleuni S Pennings, and Joachim Hermisson

Extensions of the model to include (A) linkage, (B) alternative starting frequencies, (C) diploids, and (D) asymmetric loci. Detailed analysis of (E) approximations for multi-locus architectures, (F) the marginal distribution of a single locus, and (G) the dynamics of adaptation. References in this Appendix are independent of the main text.

A. Linked loci

Negative epistasis for fitness causes negative linkage disequilibrium (LD) among the selected loci. While LD can typically be ignored as long as loci are only loosely linked, this changes once recombination rates drop below a threshold (e.g. [1], p. 277). For tight linkage $r \to 0$, in particular, individuals carrying multiple mutations can no longer be formed by recombination, but require multiple mutational hits on the same haplotype. This is unlikely while mutant allele frequencies are low, which is when the relevant mutations of the adaptive process arise. By the end of the adaptive phase, the excess of single-mutant haplotypes produces strong negative LD. Nevertheless, our theory predicts that the distribution of allele frequency ratios that emerges from the early stochastic phase of the adaptive process (Eq 9 of the main text) is unaffected. This prediction is confirmed by simulations, see Fig A.1.

Fig A.2 shows the joint distribution of the major and the minor locus of a trait with $L = 2$ loci for different degrees of linkage. In all cases, the process is stopped when the proportion of remaining non-mutant individuals drops below $f_w = 0.05$. The results show that the linkage equilibrium assumption (red and blue lines) provides a good approximation as long as $r \geq s_b$. For $r < s_b$, the distributions are shifted to lower values and clear deviations become visible. The constraint on the allele frequencies at the stopping condition changes from $(1 - p_1)(1 - p_2) = f_w$ for linkage equilibrium to $p_1 + p_2 = 1 - f_w$ for complete linkage. As a consequence, the boundary between the major and minor locus distributions (red and blue) drops from $1 - \sqrt{f_w}$ to $(1 - f_w)/2$. As shown in the Mathematical Appendix (Eq M.29), we can derive an analytical approximation for the distributions for complete linkage $r = 0$. For $L = 2$, we obtain a modified Beta-distribution (black lines in the Figure)

$$\mathrm{P}^{\pm}_{f_w,\mathrm{tl}}[p|\Theta] = \frac{2(1 - f_w)^{-1}}{B[\Theta]} \left(\frac{p}{1 - f_w}\right)^{\Theta - 1} \left(1 - \frac{p}{1 - f_w}\right)^{\Theta - 1} \tag{A.1}$$

with $p \geq (1 - f_w)/2$ (resp. $p \leq (1 - f_w)/2$) for the major (minor) locus. The simulation results show that this prediction is accurate for $r \ll s_b$ (deviations for $\Theta_{bg} = 100$ are due to overshooting of the stopping condition in the last generation of our Wright-Fisher simulations).

While linkage affects the shape of the joint distribution, it does not alter its key qualitative characteristics that distinguish adaptive scenarios. In particular, the same conditions on Θ_{bg} and Θ_l apply for singularities at the boundaries of the marginal distributions. We still observe sweep-like adaptation for $\Theta_{bg} \ll 1$, adaptation by small shifts for $\Theta_{bg} \gg 1$, and a heterogeneous pattern of partial sweeps in a transition range of Θ_{bg} around 1.

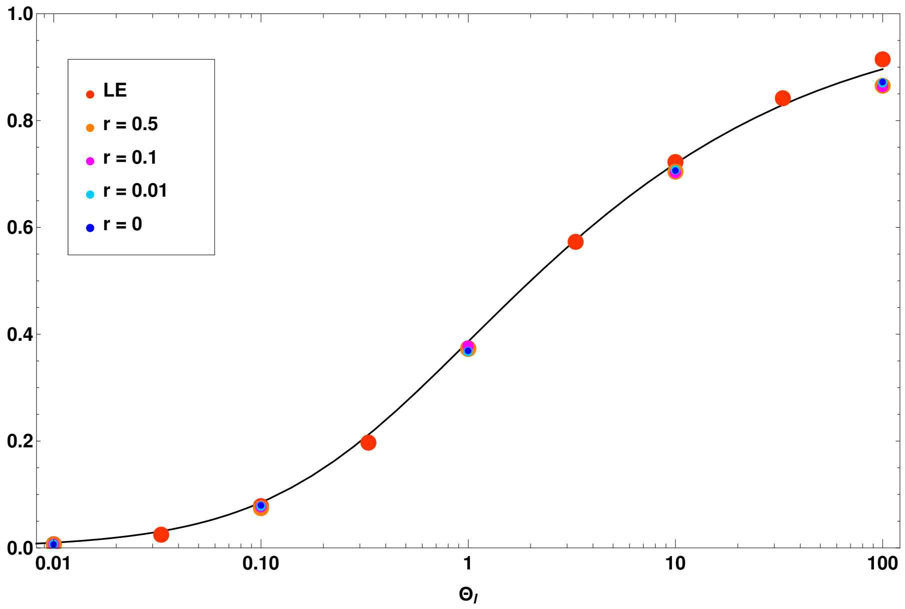

Figure A.1: E$[x]$ for redundant fitness effects with two linked loci. Simulation results (colored dots) for the mean allele frequency ratio are plotted in dependence of the locus population mutation rate Θ_l and compared with the analytical prediction (black line). Simulations are stopped when fitness has reached 95% of its maximum. Linkage does not change the results for the ratio of allele frequencies, despite significant buildup of linkage disequilibrium with low recombination rates. Results for 10 000 replicates, standard errors < 0.005 (smaller than symbols).

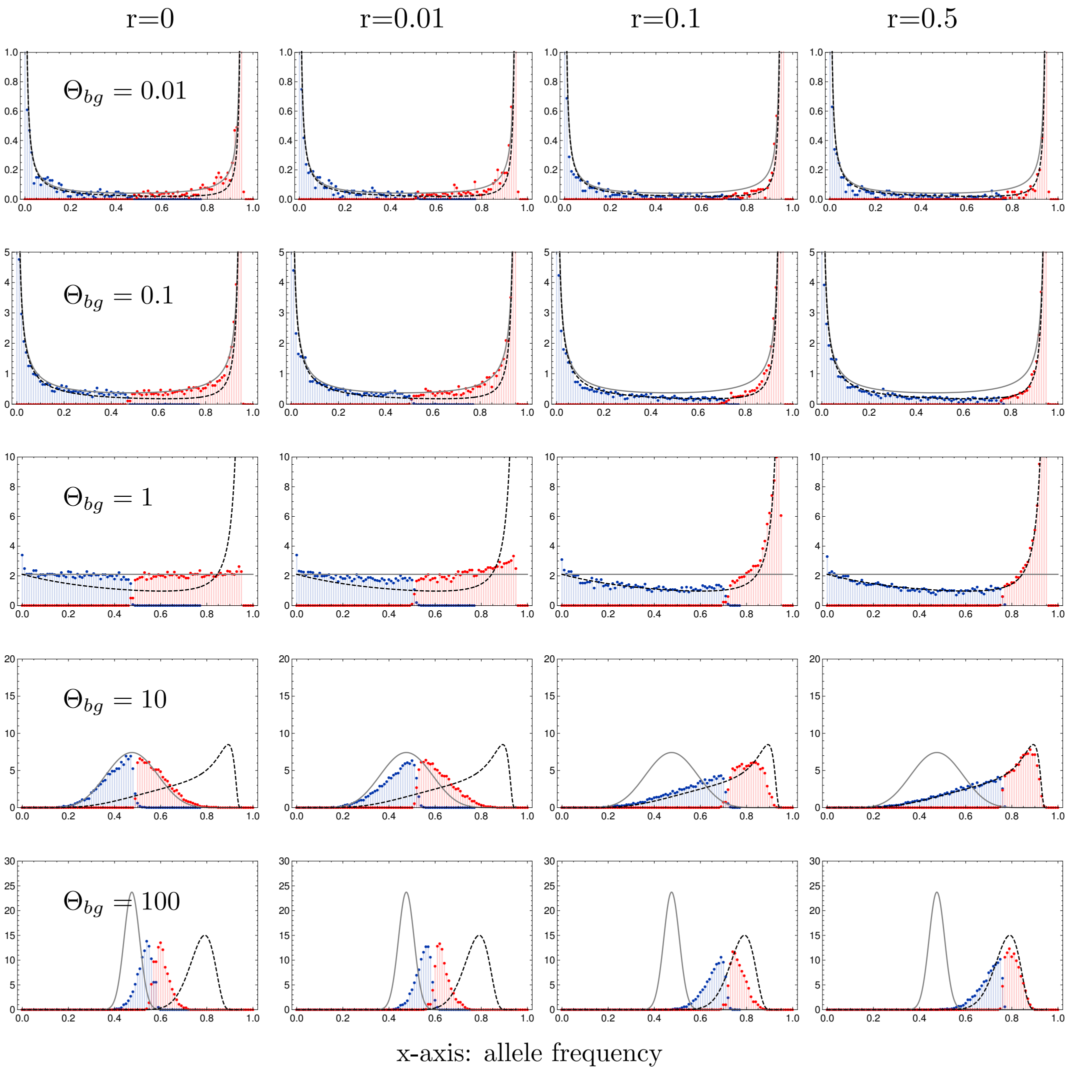

Figure A.2: Genetic architecture of adaptation with linkage. Marginal distributions for the major locus (red) and the minor locus (blue) of a model with $L = 2$ loci depending on Θ_{bg} (rows) and linkage among the loci (columns). Black lines show the analytical approximations for LE (dashed) and complete linkage (solid). For strong recombination $r \geq s_b = 0.1$, the deviations from the LE approximation are small. For $r \ll s_b = 0.1$, the approximation for complete linkage works well. Further parameters: $-s_d = s_b = 0.1$, $N_e = 10\,000$, 10 000 replicates.

B. Alternative starting allele frequencies

So far, we have assumed that adaptation starts from mutation-selection-drift balance. This includes variable amounts of standing genetic variation (weak or strong s_d) and even cases where this balance is not represented by a stable equilibrium distribution (time-dependent selection, see the Mathematical Appendix). There are, however, other

scenarios of biological relevance. Given the right (possibly complex) selection scheme, balancing selection can maintain mutant alleles, prior to the environmental change, at arbitrary frequencies. The same holds true if the base population is admixed, either due to natural processes or due to human activity (e.g. breeding from hybrids). For these scenarios, our theoretical formalism to describe the establishment of mutants during the stochastic phase (Fig 2 main text) does not apply. In this section, we describe how the formalism can be extended to cover arbitrary starting frequencies of mutants at the onset of positive selection at time $t = 0$.

Extended Yule framework

The Yule process that describes the stochastic phase of the adaptive process accounts for the mutant copies at all loci that are destined for establishment. In our framework so far (see the Mathematical Appendix, Section M.2 for details), we have started this process with zero copies. SGV due to mutation-selection-drift balance can still be produced by such a process if it is started at some time in the past ($t < 0$). For general starting frequencies, we can alternatively start this process at time $t = 0$, but with mutant copies (immortal lineages) already present. Suppose that the mutant frequency at locus i at time $t = 0$ is p_i, corresponding to $N_e p_i$ mutant copies. Of these, only the $n_i < N_e p_i$ "immortal" mutants (destined for establishment) are included in the Yule process. Assuming an independent establishment probability p_{est} per copy, n_i is binomially distributed with parameters $N_e p_i$ and p_{est}. For the limit distribution of a multi-type Yule process that is started with a non-zero number of lines, consider that each of these initial lines can be understood as an extra source of new immortal lines (due to birth) that is entirely equivalent to the generation of new lineages by mutation. It is therefore appropriate to include these lines as *extra locus mutation rate*

$$\tilde{\Theta}_i = \Theta_i + n_i = 2N_e\mu_i + n_i\,. \tag{B.1}$$

In the absence of recurrent mutation, $\Theta_i = 0$, this procedure reproduces the well-known Polya urn scheme [2, 3]. Replacing Θ_i by $\tilde{\Theta}_i$ within our original Yule process formalism, and averaging over the binomial distribution, leads to the desired extension to arbitrary starting frequencies.

Application

Theory papers (e.g. [4, 5, 6, 7]) often use a deterministic framework to describe the frequency of alleles that segregate in a population in mutation-selection balance. To simplify the analysis, they do not model SGV as a distribution (due to mutation, selection, and drift), but replace this distribution by its expected value (ignoring drift). We can apply our scheme with fixed starting frequencies to this case and thus assess the effect of genetic drift in the starting allele frequency distribution. We assume equal loci and a starting frequency $|\mu_l/s_d|$ for an (initially deleterious) mutant allele with selection coefficient s_d in mutation-selection balance. Fig B.1 shows the simulated marginal distributions of the loci with the largest contribution to the adaptive response (compare

Fig 4 main text). We see that the type of the adaptive architecture is again constant across rows with equal background mutation rate. However, due to the more homogeneous starting conditions, adaptation involves more loci and is much more shift-like. Analytical predictions following the above scheme are shown for $L = 2$ loci. With establishment probability $p_{\text{est}} = 2s_b$, the counts n_1 and n_2 of "immortal" mutants at both loci are independent random draws from a Binomial distribution with parameters $N_e|\mu_l/s_d| = |\Theta_l/2s_d|$ and $2s_b$. For $\Theta_{bg} \geq 0.1$, we find (heuristically) that the marginal distribution for alleles starting from mutation-selection balance closely matches the one of the fully stochastic model with effective $\Theta_{bg}^{\text{eff}} = \Theta_{bg}(1 + |s_b/2s_d|) = 51\Theta_{bg}$ for the parameters in the figure (lines added in green). (Note that, from the average number of established lines, one would assume $\Theta_{bg}^{\text{eff}} = \Theta_{bg}(1 + |s_b/s_d|) = 101\Theta_{bg}$. However, this does not account for the variance in the number of immortal lines among the two loci.)

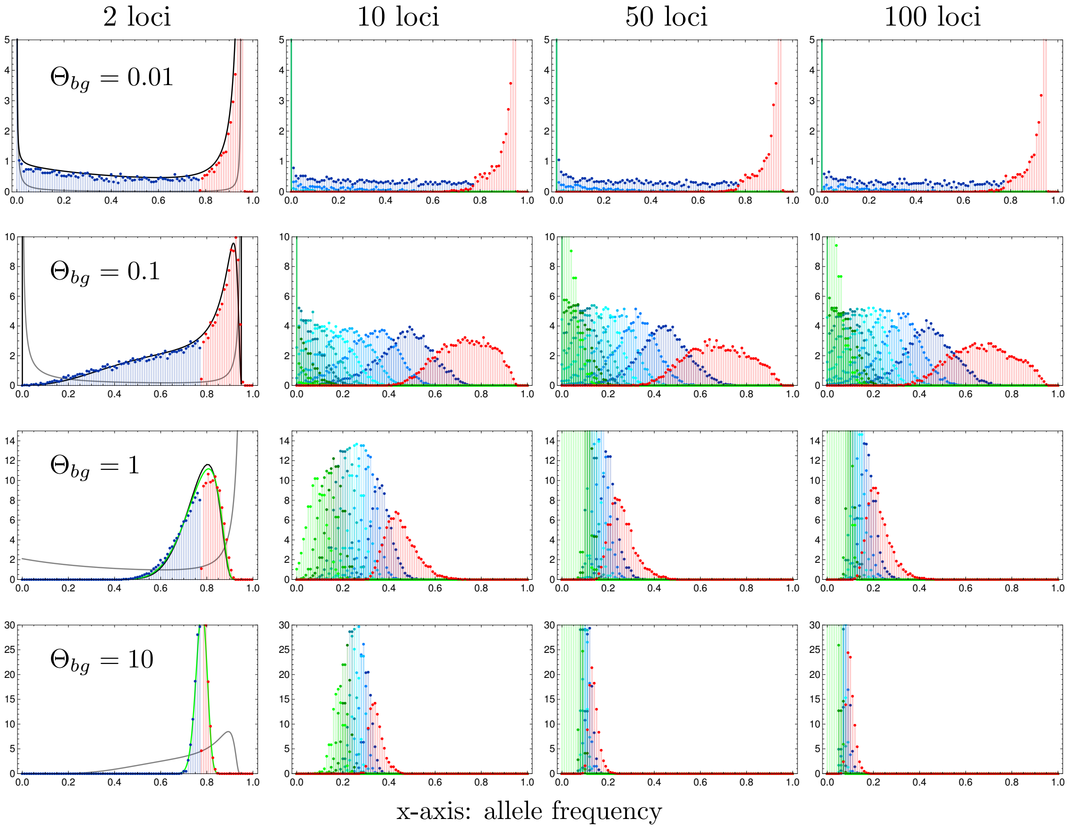

Figure B.1: Polygenic adaptation from alternative allele starting frequencies. The panels show the adaptive architecture when mutant alleles start from their expected value in mutation-selection balance, without drift. We distribute $L \cdot |\Theta_l/2s_d|$ mutant copies as evenly as possible across all loci. We set $-s_d = s_b/100 = 0.001$. Black lines for $L = 2$ loci show analytical predictions described in the main text (only computationally possible for $\Theta_{bg} \leq 1$), green lines for $\Theta_{bg} \geq 1$ show the heuristic prediction for $\Theta_{bg}^{\text{eff}} = 51\Theta_{bg}$. Finally, gray lines show the marginal distributions when adaptation occurs from mutation-selection-drift balance, compare Fig 4 main text.

C. Diploids

To extend our model to diploids, we assume that a single locus that is *homozygous* for the mutant allele is sufficient to produce the fully functional mutant phenotype, while a *heterozygous* locus produces a mutant that is functional with probability $1-h$. We assume that mutants contribute independently. Thus, if k heterozygous loci exist, but no homozygous mutant locus, the resulting mutant phenotype will be functional with probability $1-(1-(1-h))^k = 1-h^k$. For $L=2$ loci, in particular, the (logarithmic) fitness of genotype G becomes

$$w(G) = \begin{cases} 0 & \text{no mutations: } G=(aabb) \\ (1-h)s & \text{1 heterozygous locus: } G=(Aabb, aaBb) \\ (1-h^2)s & \text{2 heterozygous loci: } G=(AaBb) \\ s & \geq 1 \text{ homozygous mutation: } G=(AA.., ..BB) \end{cases}, \tag{C.1}$$

where $s = s_b > 0$ for $t \geq 0$ and $s = s_d < 0$ for $t < 0$. Note that $h \in [0,1]$ measures the dominance of the *ancestral* allele. We assume Hardy-Weinberg-linkage-equilibrium (HWLE). In this case, the marginal fitnesses of the mutant alleles are (for 2 loci),

$$w_A^* = s - (1-p_A)(1-p_B)\big[1-p_B(1-2h)\big]hs, \tag{C.2a}$$

$$w_B^* = s - (1-p_A)(1-p_B)\big[1-p_A(1-2h)\big]hs. \tag{C.2b}$$

In contrast to the haploid case, the marginal fitnesses are in general *not* equal. There are, however, two important special cases, where our fitness scheme (with redundancy on the level of loci) implies equal marginal fitnesses (and thus redundancy on the level of alleles): either if the ancestral allele is fully recessive ($h=0$) or if the alleles are co-dominant ($h=0.5$). As shown in the Mathematical Appendix, this holds true more generally for an arbitrary number of loci.

Simulation results

We simulated a diploid model with two loci in HWLE according to the above scheme with three different levels of dominance of the ancestral allele, $h = 0.1; 0.5;$ and 0.9. The diploid, effective population size is N_e, corresponding to $2N_e$ chromosomes. The mutation rate is μ at both loci and we define the population-scaled mutation rate for diploids as $\Theta_l^d = \Theta_{bg}^d = 4N_e\mu$. Simulations are stopped when the percentage of remaining ancestral *haplotypes* drops below $f_w = 0.05$. (This condition directly corresponds to the stopping condition for haploids. Alternative stopping conditions, such as 95% increase in mean diploid fitness are also covered by our theoretical framework, but require a different transformation.)

The results are shown in Fig C.1. We see that the haploid results fully carry over to diploids for co-dominance ($h=0.5$, middle column), where the diploid fitness scheme implies redundancy on the level of alleles. As explained above, the same holds true if the ancestral allele is fully recessive. Our simulations show that the haploid result is still

a good approximation for $h = 0.1$ (left column). In contrast, much larger deviations are obtained for recessive mutants (dominant ancestral allele, $h = 0.9$, right column). In this case, the locus with the higher mutant frequency experiences stronger selection. For $\Theta_l \geq 0.1$, when polymorphism at both loci is likely, this favors the major locus relative to the minor locus, increasing the heterogeneity in the adaptive architecture.

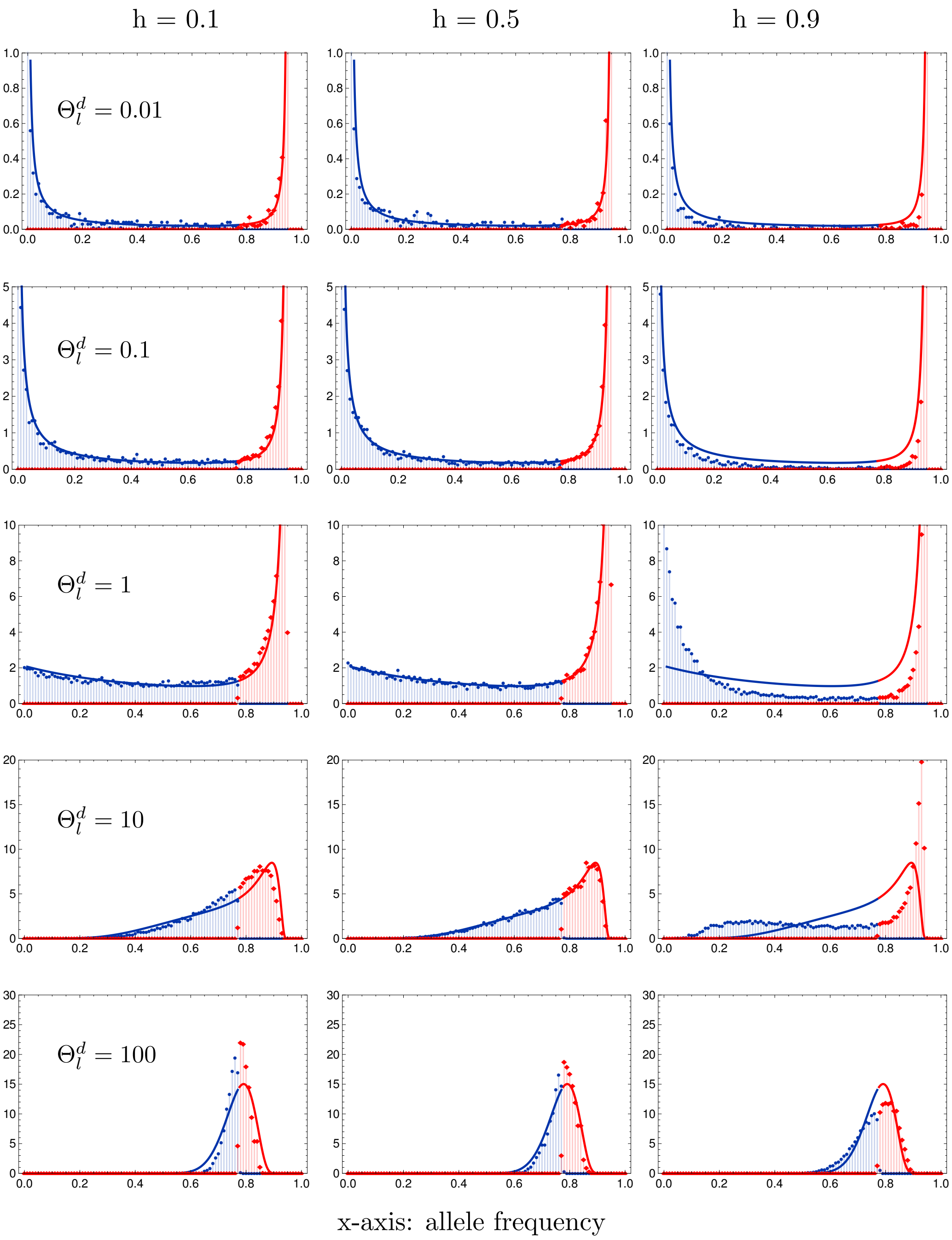

Figure C.1: Adaptive architecture for diploids in linkage equilibrium. Adaptation in a 2-locus model according to scheme (C.1), with recessive ($h = 0.1$), codomiant ($h = 0.5$) or dominant ($h = 0.9$) ancestral alleles. We assume Hardy-Weinberg and linkage equilibrium. Simulations are stopped when frequency of wildtype haplotypes drops below 5%. Standing genetic variation builds up for $16N_e$ generations before the change in the environment. Selection coefficients are set to $s_b = -s_d = 0.1$. Solid lines show analytical predictions using the framework developed for haploids.

D. Asymmetric loci

For the Figures in the main text, we have assumed that all loci in the genetic basis of the trait are equivalent: they have equal mutation rates and effect sizes. This symmetric choice favors a collective *shift* scenario because no locus has a build-in advantage. In this Appendix, we study the consequences of asymmetries among loci.

Mutation rate asymmetry Our analytical formalism allows for arbitrary asymmetries in the locus mutation rates. The prediction for the expected ratio of minor/major locus frequencies of a 2-locus model with unequal mutation rates Θ_1 and Θ_2 reads

$$\mathrm{E}[x] = \frac{\Gamma(\Theta_1+\Theta_2)}{\Gamma(\Theta_1)\Gamma(\Theta_2)} \int_0^1 \left(x^{\Theta_1-1} + x^{\Theta_2-1}\right)(1+x)^{-\Theta_1-\Theta_2}dx \tag{D.1}$$

where the sum in the integral accounts for the possibility that either locus may end up as the major locus at the time of observation (compare Eq M.27 in the Mathematical Appendix). Fig D.1 shows the prediction as a function of Θ_1 and $\Theta_2 = d\Theta_1$ together with simulation results (analogous to Fig 3 in the main text). As expected, differences in the locus mutation rates lead to more heterogeneous "sweep-like" architectures with lower minor/major locus ratio. The Figure also confirms the independence of levels of standing genetic variation and the good overall fit of the analytical approximation.

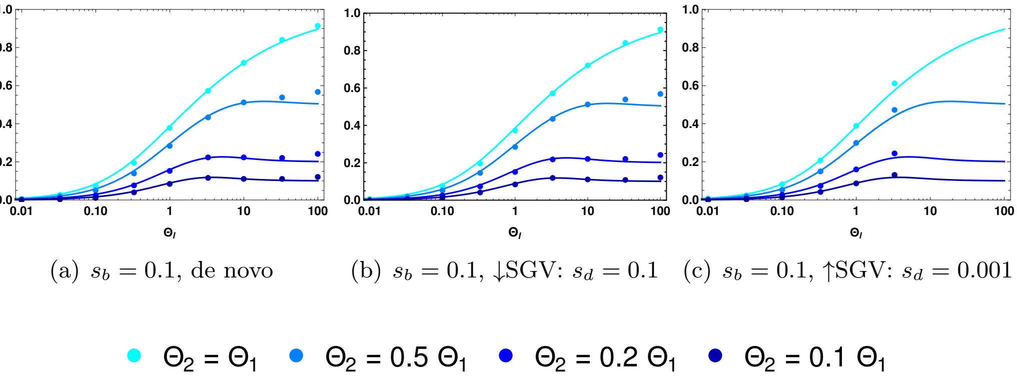

(a) $s_b = 0.1$, de novo (b) $s_b = 0.1$, ↓SGV: $s_d = 0.1$ (c) $s_b = 0.1$, ↑SGV: $s_d = 0.001$

Figure D.1: Different mutation rates. For $L = 2$ we plot $\mathrm{E}[x]$ without and with previous buildup of weak and strong SGV for different mutation rates at the two loci, such that $\Theta_2 = d{\cdot}\Theta_1$, for $d = 1, 0.5, 0.2, 0.1$. Our analytical predictions for different mutation rates, Eq (D.1), yield an excellent fit. Simulations are obtained from 10 000 replicates per data point, assuming linkage equilibrium.

Locus effect asmmetry Our analytical results are based on the assumption of strong redundancy between loci. In the main text, we have already discussed how these results extend for a scenario of relaxed redundancy, where two mutational steps are needed to reach the trait optimum. Similarly, intermediate phenotypes are also included in

the diploid version of our model. However, both model extensions do not break the symmetry assumption concerning the effects of single-locus substitutions. Differences in the single-locus effects interfere with the assumptions of our Yule-process framework for the early adaptive phase. In contrast to unequal mutation rates, they cannot easily be included. Although polygenic models with equal locus effects have a long history in the biological literature, at least slight deviations from this assumption are unavoidable in nature. Indeed, deviations already arise due to non-neutral "hitchhiker" mutations on the selected haplotypes. With exponential growth during the selected phase, even small perturbations could, in principle, lead to significant changes in the resulting adaptive architecture. To test this, we use a haploid 2-locus model with (Malthusian) fitness 0 for the ancestral genotype ab and fitness $s_{b/d} \gtrless 0$ for the single mutant Ab and the double mutant AB. The other single mutant, aB is set to $\epsilon s_{b/d}$. Fig D.2 shows simulation results for the expected minor/major frequency ratio for cases where aB is less beneficial ($\epsilon = 100/101, 10/11, 2/3$) as well as for cases where aB is optimal ($\epsilon = 101/100, 11/10, 3/2$). Note that the latter case corresponds to "sign epistasis" for the A mutant. Simulations are stopped when the frequency of ancestral haplotypes, ab, drops below 5%.

As expected, the results show that unequal locus effects (like unequal mutation rates) lead to more heterogeneous adaptive architectures. However, as long as differences in the locus effects are moderate (below $\sim 10\%$) the prediction from the fully redundant model still provides a good approximation. In contrast, differnces of 50% in the single-locus effects lead to sizable deviations. This relative robustness is reminiscent of the case of *soft selective sweeps*, where differences of $\lesssim 20\%$ in the fitness of independent mutant copies only lead to small deviations from the predictions for the frequencies of sweep haplotypes (see Fig 4 and S1 in Ref. [8]). Deviations from the fully redundant prediction are larger for the sign-epistasis case, where the aB mutant has the highest fitness. This is expected – indeed, the single mutant should eventually displace all other genotypes at later observation times. Fig D.2 also shows that deviations are partially compensated if adaptation occurs from standing genetic variation, in particular if levels of standing variation are high (panel c). This reflects our model assumption that the locus under stronger beneficial selection is also under stronger deleterious selection prior to the environmental shift.

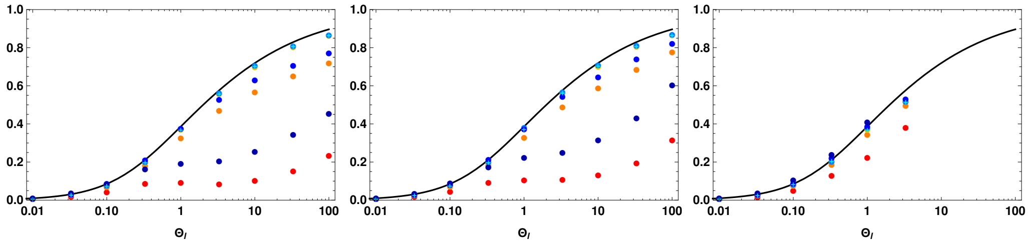

(a) $s_b = 0.1$, de novo (b) $s_b = 0.1$, ↓SGV: $s_d = 0.1$ (c) $s_b = 0.1$, ↑SGV: $s_d = 0.001$

Figure D.2: Different locus effects. For $L = 2$ we plot $\mathrm{E}[x]$ for without and with previous build-up of weak and strong SGV for various genotypic fitnesses of the aB-genotype $\epsilon s_{b/d}$. Fitness of the Ab and AB genotype is always set to $s_{b/d}$. Simulations are obtained from 10 000 replicates per mutation rate with recombination rate $r = 0.5$.

E. Approximations for multi-locus architectures

For tight linkage, where the joint distribution of mutant alleles is given by a Dirichlet distribution (Mathematical Appendix, Eq M.29), lower dimensional marginal distributions for single loci or groups of loci can easily be derived. For linkage equilibrium (Mathematical Appendix, Eq M.20), however, the required integrals can only be solved numerically. For L loci, an $(L-2)$-dim integral needs to be evaluated, which becomes computationally unfeasible (with programs packages like *Mathematica*) for $L > 5$. In many cases, we can nevertheless derive approximations. To do so, we make use of a key property of the adaptive architecture, seen in our results: The (joint) architecture of adaptation at loci with the largest contribution to the adaptive response is primarily a function of combined mutation rates at competing loci, such as the background mutation rate Θ_{bg}. Given these values, it is largely independent of the number of loci in the genetic basis of the trait itself. We can therefore describe the adaptive architecture of a polygenic trait with L loci by a model with $k < L$ loci *given that* the total adaptive response is well captured by the contribution of the top k loci. It turns out that this is typically the case for $\Theta_{bg} \leq 1$, when the contributions from different loci are very heterogeneous. In the following, we describe this procedure for an L-locus model with equal mutation rates $\Theta_i = \Theta_l$, $1 \leq i \leq L$.

Approximations using the 2-locus model

Several key properties of the L-locus architecture can already be described within the 2-locus framework. This includes the marginal distributions at the major locus and at the first minor locus. To this end, we set the mutation rate at the minor locus of the 2-locus model to the background mutation rate of the L-locus model. As described in the main text, this choice matches the time lag between the first origin of a mutation destined

for establishment at a locus (usually the major locus) and at a second locus (usually the first minor locus). It also guarantees that the approximation captures the correct asymptotic shape of the major-locus distribution at $p = 1 - f_w$, and of the first-minor-locus distribution at $p = 0$. The choice of the mutation rate at the major locus itself is less important. For the approximation of the major-locus distribution, we find that setting it to the locus-mutation rate yields the best fit. In Fig 4 in the main text, we thus use a 2-locus model with unequal mutation rates, $\mathrm{P}^{1>}_{f_w}[p_1|\Theta_l, \Theta_{bg}]$ (see Mathematical Appendix, Eq M.28a). For the marginal distribution at the first minor locus, the approximation with equal mutation rates, $\mathrm{P}^{1<}_{f_w}[p_1|\Theta_{bg}, \Theta_{bg}]$, (Mathematical Appendix, Eq M.28b), works slightly better. Finally, we can also approximate the distribution at an *average* minor locus (rather than the first minor locus) by $\mathrm{P}^{1<}_{f_w}[p_1|\Theta_l, \Theta_{bg}]$.

Approximations using models with $k \geq 2$ loci

The approximation of higher-order minor loci requires models with a sufficiently large genetic basis that such a locus exists at all. I.e., a k-locus model can approximate marginal distributions up to the $(k-1)$st minor locus. Assume that we want to approximate the marginal distribution of the jth minor locus of an L-locus model using a k-locus model, $j < k < L$. As for the case $k = 2$ discussed above, the approximation requires that the expected lag time between the origin of a successful mutation at a first locus and the origin of a mutation at a jth locus be matched. For the L-locus model, this waiting time is

$$\frac{1}{\Theta_l} \sum_{i=1}^{j} \frac{1}{L-i} \,. \tag{E.1}$$

For a k-locus model with equal mutation rate $\Theta_l^{(k)}$ at all loci, we thus obtain the matching rule

$$\Theta_l^{(k)} = \Theta_l \, \frac{\sum_{i=1}^{j} \frac{1}{k-i}}{\sum_{i=1}^{j} \frac{1}{L-i}} \tag{E.2}$$

for the approximation of the jth minor locus. For $j = 1$, this reproduces the matching rule for the background mutation rate Θ_{bg}. In general, the value for $\Theta_l^{(k)}$ depends on j, but converges once $L, k \gg j$. Approximations by models with unequal locus mutation rates are also possible, but usually do not lead to a relevant improvement. In Fig 4 in the main text, we use formulas from 3- and 4-locus models to approximate the marginal distributions of the 2nd and 3rd minor locus, respectively. In general, the approximations for all loci can be improved by using approximation models with more loci than required, i.e. $k > j + 1$. In main text Fig 4, we show this for approximations of the major locus and the first three minor loci, all derived from a 4-locus model.

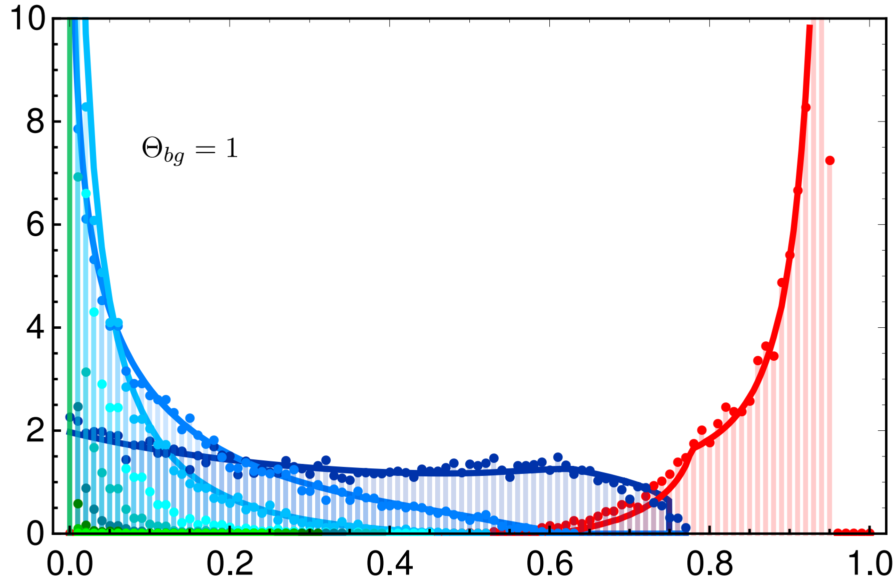

Figure E.1: Approximating higher dimensional adaptive architectures. We approximate a 10 locus model ($\Theta_{bg} = 1$) with the theoretical predictions based on the four-locus model for the major locus and the first, second, and third minor locus. Compare Fig 4 in the main text, where we use approximations based on models with the minimal number of loci needed.

F. Marginal distribution of a single locus

Figure F.1 shows the marginal distribution at a single focal locus for a trait with $L = 2$ to $L = 100$ loci in its basis. Since all loci are equal, the probability that the focal locus ends up as the major locus is $1/L$. The red dots in the figure indicate the part of the marginal distribution that corresponds to this case. With an increasing number of redundant loci, the probability for each single locus to play a major role in the adaptive process decreases. The marginal distribution of a fixed locus therefore changes strongly with an increasing number of loci L. For large L, in particular, it does not represents the key components of the adaptive architecture on the level of the trait any more. This is in contrast to Fig 4 in the main text, where marginal distributions of the loci with the largest contributions to the adaptive response are shown. For 2 loci, Fig F.1 also shows the analytical approximation for the marginal distribution (Eq 11 main text). As long as the adaptive architecture is dominated by only a few loci, the same 2-locus result can be used as an approximation for the marginal distribution in models with more than two loci. This is shown in the figure for $\Theta_{bg} \leq 1$. The figure also shows that the approximation fails for $\Theta_{bg} \geq 10$ when adaptation is truly collective.

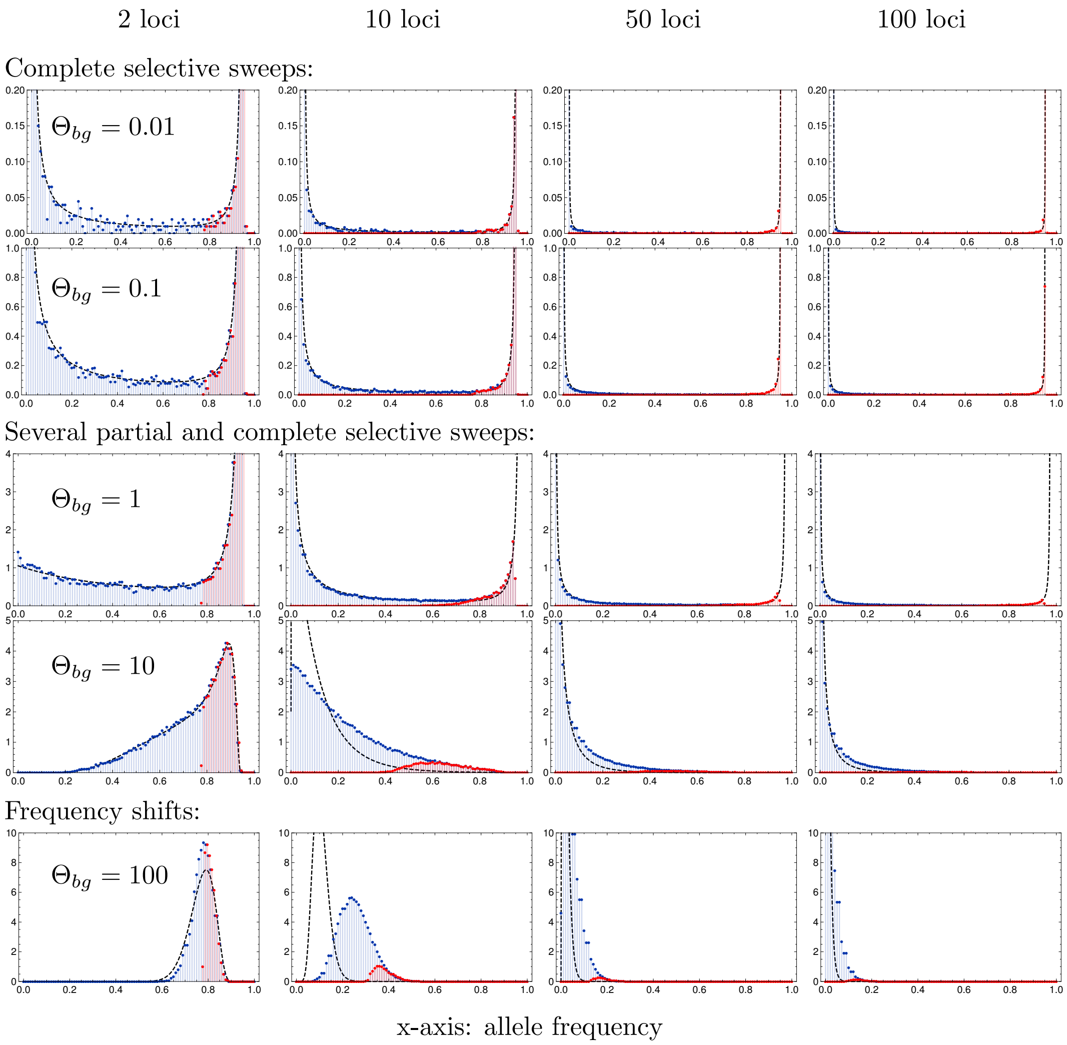

Figure F.1: Marginal distribution at a single focal locus. Simulation results for the marginal distribution at a single locus at the end of the adaptive phase are shown in blue. Red dots show the contribution of the major locus to this distribution (all cases, where the focal locus ends up as the major locus). Dashed lines show the analytical prediction based on the 2-locus model (Eq 11 main text). Parameters and further details as in main text Fig 4.

G. Dynamics of adaptation

In contrast to previous work on the topic (e.g. [6, 7]), our approach does not discuss adaptive architecture as a function of the time that has elapsed since the environmental change. Instead, we assess adaptation at the genotypic level as a function of the progress that has been made towards adaptation of the trait. In our main result on the joint distribution of mutant allele frequencies (Eq 8 in the main text), this progress is measured by the stopping condition f_w, which directly relates to the distance of the trait mean to the new optimum (for the basic model of a fully redundant trait, f_w is the frequency of remaining ancestral phenotypes in the population, see Eq 2 main text). This shift from a time-slice view to a trait-centered view can lead to larger qualitative differences in particular if the mutation rate is low ($\Theta_l \ll 1/L$). In this case, a distribution of genetic architectures at a fixed time $t > 0$ will incorporate opposite cases where adaptation of the trait has either already been completed or not even started because the population still waits for a successful mutant. Biologically, a trait-centered view seems to be closer to the idea of an "architecture of phenotypic adaptation". Mathematically, the changed perspective enables the derivation of analytical results. By comparing architectures for variable degrees of phenotypic adaptation, we still obtain a view of the adaptation dynamics, with f_w as dynamical variable instead of time t. This is shown in Fig G.1. For $\Theta_{bg} \leq 1$, we see how the dominant contribution of a single "major locus" to the adaptive response emerges early on and then accentuates during the adaptive phase.

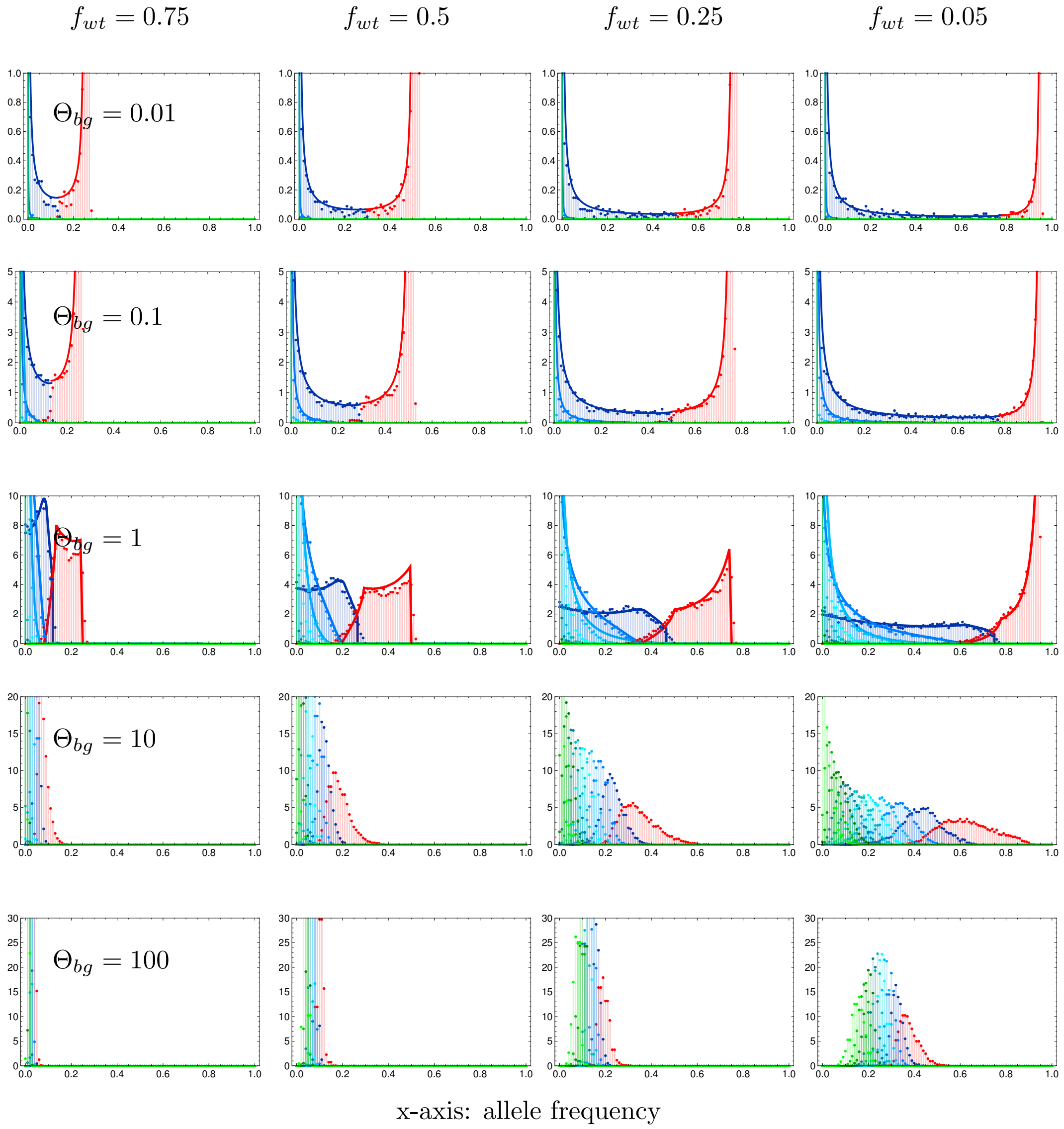

Figure G.1: Dynamics of the adaptive process. Allele frequency distributions at four stages over the course of adaptation. Approximations correspond to the Fig E.1 each rescaled to the changed stopping condition $f_{wt} = 0.75; 0.5; 0.25; 0.05$. Simulations for 10 000 replicates per mutation rate with $s_b = -s_d = 0.1$.

References

1. Bürger R. The mathematical theory of selection, recombination, and mutation. Wiley, Chichester, UK; 2000.

2. Griffiths R, Tavaré S. The age of a mutation in a general coalescent tree. Stochastic Models. 1998;14(1-2):273–295.

3. Hoppe FM. Pólya-like urns and the Ewens' sampling formula. Journal of Mathematical Biology. 1984;20(1):91–94.

4. Orr HA, Betancourt AJ. Haldane's sieve and adaptation from the standing genetic variation. Genetics. 2001;157(2):875–884.

5. de Vladar HP, Barton NH. Stability and response of polygenic traits to stabilizing selection and mutation. Genetics. 2014;197(2):749–767.

6. Jain K, Stephan W. Response of polygenic traits under stabilizing selection and mutation when loci have unequal effects. G3: Genes, Genomes, Genetics. 2015;5(6):1065–1074.

7. Jain K, Stephan W. Rapid adaptation of a polygenic trait after a sudden environmental shift. Genetics. 2017;206(1):389–406.

8. Pennings PS, Hermisson J. Soft sweeps II–molecular population genetics of adaptation from recurrent mutation or migration. Molecular Biology and Evolution. 2006;23(5):1076–1084.
