# Supplementary material for: Polygenic adaptation: From sweeps to subtle frequency shifts
Source: PLoS Genet. 2019 Mar 20;15(3):e1008035. doi: 10.1371/journal.pgen.1008035 (PMC6443195; doi:10.1371/journal.pgen.1008035)
Supplement: S2 Appendix — Detailed description and derivation of the analytical results. (PDF) [file pgen.1008035.s002.pdf]

S2 Appendix for *Polygenic adaptation: From sweeps to subtle frequency shifts*

Ilse Höllinger, Pleuni S Pennings, and Joachim Hermisson

Mathematical Appendix

This Appendix describes the details of the mathematical model and methods used to derive the analytical results of the article. Section M.1 gives an outline of the model; section M.2 introduces the branching process method used for the early stochastic phase of polygenic adaptation; section M.3 describes the derivation of the joint frequency distribution at the end of the deterministic phase. The list of references at the end of the document is independent from the main text.

M.1 Redundant trait model

Consider a panmictic population of N_e haploids. Selection acts on a binary trait Z (e.g. resistance) with just two states, a wildtype state Z_0 (not resistant) and a mutant state Z_1 (resistant). Without restriction, we can choose $Z_0 = 0$ and $Z_1 = 1$. Malthusian (logarithmic) fitness is defined by the function

$$W(Z,t) = s(t)Z \tag{M.1}$$

where the time dependent coefficient $s(t)$ defines the strength of directional selection. We assume that $s(t) < 0$ for $t < 0$, but $s(t) > 0$ for $t > 0$, such that the optimal trait value shifts from the wildtype state $Z = 0$ to the mutant state $Z = 1$ due to some change in the environment at time $t = 0$. We also assume that selection is stronger than drift, $|Ns(t)| \gg 1$ for almost all t, but is arbitrary otherwise.

We assume that Z is polygenic, with L biallelic loci (wildtype a_i and mutant allele A_i, $i = 1, \ldots, L$) constituting its genetic basis. While genotype $\mathbf{a} = (a_1, a_2, \ldots, a_L)$ produces the ancestral wildtype Z_0, all mutant genotypes are fully redundant and produce the mutant phenotype Z_1, independently of the number of mutations. New mutations from a_i to A_i occur at a rate μ_i per generation, with $\mu_i \ll |s(t)|$ for almost all t. For the purpose of our model, back mutation from A_i to a_i can be ignored. The linkage map among loci is arbitrary – unless explicitly specified otherwise. Let p_i be the frequency of allele A_i, and let f_a be the frequency of the wildtype genotype $\mathbf{a}$. Then the mean fitness in the population is

$$\bar{W}(t) = s(t)\bar{Z}(t) = s(t)\left(f_a Z_0 + (1 - f_a)Z_1\right) \tag{M.2a}$$

where $\bar{Z}$ is the trait mean. Since $W(Z_1, t) = s(t)Z_1$ is the marginal fitness of any mutant allele, the selection dynamics at the ith locus can be expressed as

$$\dot{p}_i = p_i\big(W(Z_1, t) - \bar{W}(t)\big) = s(t)p_i\big(Z_1 - \bar{Z}(t)\big)\,. \tag{M.2b}$$

Our redundancy assumption implies strong diminishing returns epistasis on the level of fitness: the fitness of genotypes with multiple mutations is the same as the one of single mutants. Eq (M.2b) shows that the epistatic effect of the genetic background on the dynamics at a particular locus is mediated by the trait mean $\bar{Z}(t)$ as single compound parameter. Allele frequencies at all loci change with the same (time and frequency-dependent) rate. We readily establish that

$$\frac{d}{dt}\left(\frac{p_i}{p_j}\right) = \frac{\dot{p}_i p_j - \dot{p}_j p_i}{p_j^2} = 0\,. \tag{M.3}$$

Thus, the ratio of allele frequencies among loci does not change under selection. Note that this holds for an arbitrary linkage map. We can conclude that any differences in (relative) allele frequencies are due to mutation and drift.

We are interested in the pattern of allele frequency changes across loci during the phase of rapid phenotypic adaptation. This phase starts with the onset of positive selection on derived alleles at time $t = 0$. It ends when mean fitness $\bar{W}(t)$ approaches its maximum $s(t)Z_1$ and further selective change in the allele frequencies is strongly decelerated. Since $(W(Z_1, t) - \bar{W}(t))/s(t) = (Z_1 - Z_0)f_a$, we can parametrize this end point by a condition $f_a(t) = f_w$ on the frequency of the wildtype Z_0 in the population. In our figures, we usually use $f_w = 0.05$. As initial state at time $t = 0$, we assume that the population adapts from a balance of mutation, selection, and drift. We thus allow for standing genetic variation (SGV) at all loci. If selection prior to $t = 0$ is constant (which is what we generally assume in our computer simulations, see main text), SGV is given by the standard equilibrium distribution under mutation, selection, and drift, where we require that a_i is the ancestral state at each locus. I.e., each allele frequency trajectory $p_i(t)$, back in time, originates from the boundary $p_i = 0$ rather than $p_i = 1$ (see also [1] for this concept). However, our analytical results do not require a static equilibrium and, for a general $s(t) < 0$ for $t < 0$, the SGV reflects this non-equilibrium dynamics.

As described in the main text, we dissect the adaptive process into two phases. During an initial *stochastic phase* mutation, selection, and drift lead to the build-up of genetic variation, either from SGV or due to new mutation after time $t = 0$, as long as allele frequencies p_i at all loci are still low. We will describe our approach to this phase in detail in the section on Yule processes below. Once allele frequencies are sufficiently large, genetic drift and recurrent new mutation play only a minor role relative to selection until we reach the end of the rapid adaptive phase. We thus enter a *deterministic phase* where the dynamics is then well approximated by Eq (M.2b).

Relaxed redundancy

To relax the stringent redundancy condition of our model, it is natural to assume that a single mutation is not sufficient to produce the full mutant phenotype $Z_1 = 1$, but only a partial phenotype $Z_q = q$ with $0 < q < 1$. This makes the marginal fitness of mutant alleles dependent on the genetic background. If genotypes with two or more mutations

produce Z_1, we have

$$\dot{p}_i = \big(W_i(t) - \bar{W}(t)\big)p_i = s(t)p_i\bigg(Z_1 - \bar{Z}(t) - (Z_1 - Z_q)\frac{f_i}{p_i}\bigg) \tag{M.4}$$

where f_i is the frequency of the haplotype with a single mutation at locus i. Since f_i/p_i depends on i (even in linkage equilibrium), the ratio of allele frequencies at different loci is no longer invariant and the key symmetry assumption (M.3) of the fully redundant model is violated. Note that redundancy is recovered for very low mutant frequencies, such that double mutants are rare ($f_i \approx p_i$) and also late in the adaptation process, when most haplotypes carry at least one mutation and $f_i \to 0$.

Diploids

We can generalize the redundant trait model to diploids as follows. For a general model, the dynamical equations in continuous time read

$$\dot{p}_i = \big(W_i(t) - \bar{W}(t)\big)p_i \tag{M.5}$$

where $W_i(t)$ is the marginal fitness of allele A_i and $\bar{W}(t)$ the mean fitness. All fitnesses may depend on the allele frequencies and on time. Using (M.3), we see that all mutant alleles A_i are redundant in the sense that they all feel the same selection pressure if and only if their marginal fitnesses are equal at all times, $W_i(t) = W_j(t)$, $\forall\ i, j$. (The same condition can also be derived from a discrete time dynamics.) For haploids, equal marginal fitnesses, independently of the genetic composition of the population, enforces the fully redundant trait model described above. For diploids with dominance, the marginal fitness also depends on the allele frequency at the focal locus itself. An obvious solution to the condition of equal marginal fitnesses across loci is the case of complete dominance of the mutant allele. We can gain some more flexibility for the fitness scheme, if we assume that genotype frequencies are at Hardy-Weinberg equilibrium at all times. We can then distinguish three genotype classes: the wildtype without any mutations (normalized fitness 0), mutant individuals with one or more mutations on only a single haplotype (fitness $s_1(t)$) and individuals with mutations on both haplotypes (fitness $s_2(t)$). The marginal fitness of any mutant allele then is

$$W_i(t) = s_1(t)f_a + s_2(t)(1 - f_a)\,, \tag{M.6}$$

where f_a is the frequency of the ancestral haplotype without mutations. We thus require redundancy of mutations (only) within haplotypes. Note, however, that this fitness scheme implies a position effect, i.e., the fitness of the genotype does not only depend on the number of mutations at each locus, but also on the association of mutations to one or the other haplotype. If we assume linkage equilibrium in addition to Hardy-Weinberg proportions, a position effect can be avoided if we use the following fitness scheme

1. The ancestral genotype without any mutants has normalized fitness $W(t) = 0$,
2. any genotype with at least one homozygous mutant has fitness $W(t) = s_2(t)$,

3. a genotype without a locus that is homozygous for the mutant, but with k loci that are heterozygous has fitness

$$W(t) = s_2(t) + 2^{1-k}\Big(s_1(t) - s_2(t)\Big)\,.$$

Since 2^{1-k} is the probability for any focal mutant allele to be on the same haplotype with all $k-1$ other mutant alleles, assuming linkage equilibrium, this fitness scheme leads to the same marginal fitness as Eq (M.6) above.

M.2 Yule approximation

We describe the dynamics of mutant types at the different loci during the stochastic phase by a *multi-type Yule pure birth process with immigration.* Our framework builds on established mathematical theory [2, 3] and a previous approach to describe the genealogy of a beneficial allele during a selective sweep in terms of a Yule process [4, 5]. Here, we extend this approach to the polygenic scenario.

Consider a mutation A_i that appears at some locus either prior to the environmental change (standing genetic variation) or after the change. This mutation is relevant for the joint distribution of mutant allele frequencies at the time of observation after the rapid adaptive phase if and only if descendants of this mutation still segregate in the population at this time. The idea of the Yule approach is to construct the genealogies of these mutant descendants at all loci forward in time. We start the process at some time $t_0 \ll 0$ in the past before the first mutation with surviving descendants has originated. We assume that the frequency p_i of mutant alleles is low during the entire stochastic phase. Then, new mutations at locus i appear at rate $\approx N\mu_i =: \Theta_i/2$ per generation, but only a fraction of those will survive deleterious selection prior to $t = 0$ and genetic drift to establish in the population and to contribute to the adaptation of the trait. We denote this establishment probability as $p_{\text{est}}(t)$. If selection is constant and positive (as assumed in the main text), $s(t) = s_b > 0$, we can approximate $p_{\text{est}} \approx 2s_b$. For general time-dependent selection, $p_{\text{est}}(t)$ will depend on $s(\tilde{t})$ with $\tilde{t} \geq t$ [6], and also on the mutations that were previously established at the same or at other loci. Crucially, however, since the marginal fitness of mutant copies at all loci is the same at any given time, $p_{\text{est}}(t)$ does not depend on the locus. We only include mutants into our Yule process that successfully establish in the population, which are represented as "immortal lineages" in the Yule tree. We follow these lineages in continuous time. There are then two types of events:

1. First, new mutation creates new immortal lineages at rate

$$p_{\text{mut},i}(t) = \frac{\Theta_i}{2}\, p_{\text{est}}(t) \tag{M.7}$$

independently at each locus. This event is called "immigration" in the mathematical literature [2], but it corresponds to mutation in our model. (In a model with gene flow, where adaptation in a local deme occurs from immigration, new lines would be truly immigrants, see also [7] for this analogy).

2. Second, existing immortal mutant alleles A_i can give birth to further immortal mutant copies, corresponding to a split of the immortal line in the Yule process. To derive the split rate p_{split}, imagine that we implement the evolutionary dynamics as a continuous-time Moran model, where individuals give birth (due to a binary split) at constant rate one per generation. In the corresponding Yule process, we only include this birth event if it leads to two immortal lineages. Obviously, the probability to "be immortal" for a newborn individual is the same as for a new mutation and given by $p_{\text{est}}(t)$. Conditioning on the fact that we only consider splits of immortal lineages and thus at least one of the offspring lineages must be immortal, we arrive at a split rate per immortal lineage of

$$p_{\text{split}}(t) = \frac{p_{\text{est}}^2(t)}{p_{\text{est}}^2(t) + 2p_{\text{est}}(t)(1 - p_{\text{est}}(t))} = \frac{p_{\text{est}}(t)}{2 - p_{\text{est}}(t)} \approx \frac{p_{\text{est}}(t)}{2}\,, \tag{M.8}$$

where the approximation in the last term assumes that $p_{\text{est}}(t) \ll 1$, which is usually the case unless selection is very strong.

The Yule process defines a continuous-time Markov process of a random variable $\mathbf{k} = (k_1, \ldots, k_L)$, where $k_i \in \mathbb{N}_0$ is the number of immortal mutant lineages at the ith locus. We are interested in the relative proportions in the number of lineages k_i across loci after a sufficiently long time – assuming that the distribution of these proportions reaches a limit by the end of the stochastic phase. We can generate this distribution from the transition probabilities among Yule states (the embedded jump-chain of the continuous-time process). If there are currently $(k_1, \ldots, k_L)$ lineages at the L loci, the probability that the next event is either a birth event (split) or a new mutation (immigration) at locus i is

$$\begin{aligned}\Pr[(k_1, \ldots, k_L) \to (k_1, \ldots, k_i + 1, \ldots, k_L)] \\ = \frac{k_i p_{\text{split}} + p_{\text{mut},i}}{\sum_{j=1}^{L}(k_j p_{\text{split}} + p_{\text{mut},j})} = \frac{k_i + \Theta_i}{\sum_{j=1}^{L}(k_j + \Theta_j)}\,.\end{aligned} \tag{M.9}$$

Crucially, these transition probabilities are constant in time and independent of the establishment probability $p_{\text{est}}(t)$. As a consequence, they are also independent of the mutant fitness, which only affects the speed of the Yule process (via p_{est}), but not its sequence of events.

We start the process with no mutants and stop it whenever the number of mutants at one of the loci (e.g. locus 1) reaches some number $k_1 = n$. We are interested in the distribution of the number of mutants k_i at the other loci at this time, respectively their ratios k_i/n (remember that we already know that these ratios stay invariant during the deterministic phase of the adaptation process). We can prove the following

Theorem 1 In the limit of $n \to \infty$, the joint distribution of ratios $x_i = k_i/n$ of immortal mutant lineages across loci converges to the *inverted Dirichlet distribution*,

$$\mathrm{P}_{\text{inDir}}[\{x_i\}_{i\geq 2}|\boldsymbol{\Theta}] = \frac{1}{B[\boldsymbol{\Theta}]} \prod_{j=2}^{L} x_j^{\Theta_j - 1}\Big(1 + \sum_{j=2}^{L} x_j\Big)^{-\sum_{j=1}^{L}\Theta_j} \tag{M.10}$$

where the vector $\mathbf{\Theta} = (\Theta_1, \ldots, \Theta_L)$ summarizes the mutation rates and $B[\mathbf{\Theta}]$ is the multivariate Beta function, which can be expressed in terms of Gamma functions as

$$B[\mathbf{\Theta}] = \frac{\prod_{i=1}^{L} \Gamma(\Theta_i)}{\Gamma(\sum_{i=1}^{L} \Theta_i)} . \tag{M.11}$$

Proof We proceed in three steps.

Step 1 Assume that we stop the process when the first locus reaches $n > 0$ lineages. We derive the probability that the process at this time is in state $(n, k_2, \ldots, k_L)$ as follows. We need $n+k_2+\cdots+k_L$ events (new mutations or splits) to generate all mutant individuals. The last event must occur at the first locus. All other events can occur in arbitrary order at the L loci. The probability of each realization (each order of events at the loci) is given by the corresponding product of transition probabilities (M.9). The key insight is that all realizations have the same probability. Indeed, the denominator of (M.9) does not depend on the locus where the next event occurs. Different realizations then only correspond to permutations in the factors $k_i + \Theta_i$ in the numerator of the product of transition probabilities. We can directly write down the probability for the state as

$$\Pr[\{k_i\}_{i\geq 2}|n, \mathbf{\Theta}] = \binom{n-1+k_2+\cdots+k_L}{n-1, k_2, \ldots, k_L} \frac{(\Theta_1)_{(n)} \prod_{j=2}^{L} (\Theta_j)_{(k_j)}}{(\Theta_1 + \cdots + \Theta_L)_{(n+k_2+\cdots+k_L)}}, \tag{M.12}$$

where

$$\Theta_{(k)} := \Theta(\Theta+1)\ldots(\Theta+k-1)$$

is the Pochhammer function. The leading multinomial coefficient counts the number of all permutations and the ratio of Pochhammer functions is the probability of each realization.

Step 2 We can rewrite (M.12) as a *Dirichlet-negative-multinomial* compound distribution, defined as

$$\int_0^1 \cdots \int_0^1 \binom{n-1+k_2+\cdots+k_L}{n-1, k_2, \ldots, k_L} \prod_{i=2}^{L} y_i^{k_i} \Big(1 - \sum_{i=2}^{L} y_i\Big)^n f(\{y_i\}_{i\geq 2}|\mathbf{\Theta})\, dy_2 \ldots dy_L \,, \tag{M.13}$$

where

$$f(\{y_i\}_{i\geq 2}|\mathbf{\Theta}) = \frac{1}{B[\mathbf{\Theta}]} \prod_{i=2}^{L} y_i^{\Theta_i - 1} \Big(1 - \sum_{i=2}^{L} y_i\Big)^{\Theta_1 - 1}$$

is the $(L-1)$-dimensional Dirichlet distribution for a L-dimensional probability vector $(y_1, \ldots, y_L)$ with constraint $y_1 = 1 - \sum_{i\geq 2} y_i$. This is best shown in the reverse direction, i.e., by deriving (M.12) from (M.13). To see this, note that

$$\int_0^1 \cdots \int_0^1 \prod_{i=2}^{L} y_i^{\Theta_i+k_i-1}\Big(1-\sum_{i=2}^{L} y_i\Big)^{\Theta_1+n-1} dy_2\dots dy_L = \frac{\Gamma(\Theta_1+n)\prod_{i=2}^{L}\Gamma(\Theta_i+k_i)}{\Gamma\big(\Theta_1+n+\sum_{i=2}^{L}(\Theta_i+k_i)\big)}$$

because the integrand in this expression is just a Dirichlet density with shifted values of $\Theta_i \to \Theta_i + k_i$ and the right hand side is the corresponding normalization factor. Then using

$$\frac{\Gamma(\sum_{i=1}^{L}\Theta_i)}{\prod_{i=1}^{L}\Gamma(\Theta_i)}\frac{\Gamma(\Theta_1+n)\prod_{i=2}^{L}\Gamma(\Theta_i+k_i)}{\Gamma\big(\Theta_1+n+\sum_{i=2}^{L}(\Theta_i+k_i)\big)} = \frac{(\Theta_1)_{(n)}\prod_{j=2}^{L}(\Theta_j)_{(k_j)}}{(\Theta_1+\cdots+\Theta_L)_{(n+k_2+\cdots+k_L)}}$$

reduces (M.13) to (M.12).

The compound distribution Eq (M.13) can be interpreted as follows: If a random experiment can have a finite number of outcomes (here: mutant lineages at one of L loci), the negative multinomial distribution describes the probability to observe each of these events k_i times if we repeat the experiment until a focal event (here: new mutant lineage at the first locus) has occurred n times. While the negative multinomial distribution assumes that all outcomes occur with a fixed probability y_i, this probability is itself drawn from a Dirichlet distribution in the Dirichlet-negative-multinomial compound distribution. In the present context, the main advantage of (M.13) over (M.12) is that we can easily perform the limit $n \to \infty$ in this form.

Step 3 For large $n \to \infty$, the values of k_i/n, $i \geq 2$, of the negative multinomial distribution can be replaced by their expectations,

$$x_i := \mathrm{E}\left[\frac{k_i}{n}\right] = \frac{y_i}{1-\sum_{j=2}^{L} y_j} \;\Leftrightarrow\; y_i = \frac{x_i}{1+\sum_{j=2}^{L} x_j}\,.$$

We can then transform the density (M.10) from variables y_i to the x_i (representing the relative mutant frequencies). The entries of the Jacobian matrix (for $2 \leq i, j \leq L$) are

$$\mathbf{J}_{ij} = \frac{\partial y_i}{\partial x_j} = \frac{\delta_{i,j}(1+\sum_{k=2}^{L} x_k) - x_i}{(1+\sum_{k=2}^{L} x_k)^2}\,.$$

Since this is the sum of an identity matrix (times a factor) and a matrix with identical columns we can easily derive the eigenvalues and thus the determinant,

$$\mathrm{Det}[\mathbf{J}] = \frac{1}{(1+\sum_{k=2}^{d} x_k)^L}\,.$$

Applying this transformation to (M.13), we obtain (M.10).

Remarks

1. For two loci, the Dirichlet-negative-multinomial distribution (M.13) reduces to a *Beta-negative-binomial* distribution

$$\mathrm{P}_{\beta NB}[k|n] = \int_0^1 \binom{n+k-1}{k} y^k (1-y)^n \frac{\Gamma(\Theta_1+\Theta_2)}{\Gamma(\Theta_1)\Gamma(\Theta_2)}\, y^{\Theta_2-1}(1-y)^{\Theta_1-1}\, dy$$

   and the inverted Dirichlet distribution (M.10) simplifies to a so-called *β-prime* distribution,

$$\mathrm{P}_{\beta'}(x) = \frac{\Gamma(\Theta_1+\Theta_2)}{\Gamma(\Theta_1)\Gamma(\Theta_2)}\, x^{\Theta_2-1}\big(1+x\big)^{-\Theta_1-\Theta_2}\,. \qquad \text{(M.14)}$$

   If we measure the ratio x always relative to the locus with the higher frequency, we obtain a conditioned distribution that is truncated at $x = 1$. For equal locus mutation rates $\Theta_1 = \Theta_2 = \Theta_l$, in particular,

$$\mathrm{P}_{\beta'}[x|\Theta_l] = \frac{2\Gamma(2\Theta_l)}{(\Gamma(\Theta_l))^2}\, x^{\Theta_l-1}(1+x)^{-2\Theta_l}. \qquad \text{(M.15)}$$

   with expectation

$$\mathbf{E}[x] = \int_0^1 x P_{\beta'}[x|\Theta_l]dx = \frac{2\Gamma(2\Theta_l)\, {}_2F_1[2\Theta_l, 1+\Theta_l, 2+\Theta_l, -1]}{(1+\Theta_l)(\Gamma(\Theta_l))^2}\,, \qquad \text{(M.16)}$$

   where ${}_2F_1$ is the hypergeometric function.

2. The process described here is a variant of the *Polya urn* and *Hoppe urn* processes that are well-known in the mathematical literature and have been used to describe coalescent processes forward in time [2, 3].

3. Our result (M.10) can also be seen as multi-locus version of Wright's formula for the stationary distribution of the Wright-Fisher diffusion [8]. For L neutral alleles at a singe locus, and if the mutation rates Θ_i depend only on the target allele (house-of-cards condition), this is a Dirichlet distribution. Here, we see that an analogous result holds for a distribution of equivalent (mutually redundant) alleles across L loci. Although alleles at different loci cannot mutate into each other and are never identical by descent, it turns out that the genealogy in both models can be described by a Yule process with immigration. In contrast to the single-locus case, we obtain an *inverted* Dirichlet distribution for multiple loci. This difference results from a different stopping condition for the Yule process. For a single locus, the population size sets an upper bound for the total number of copies across all alleles. If we stop the process for a given total number n_{tot} of lines, we obtain the classical Dirichlet distribution in the limit $n_{\text{tot}} \to \infty$. In contrast, the population size defines a bound for mutants of a only single type in the multi-locus case, which is reflected by our choice of the stopping condition. This choice is appropriate unless all loci are tightly linked, as we will see below.

4. In our model, we did not distinguish different mutational origins of mutant alleles at the same locus. It is, in principle, possible to do so. For any single locus, the process *conditioned on* reaching some number of mutants k_i at this locus i is entirely independent of the process at the other loci. The joint distribution of different mutational origins at this locus is therefore given by the Ewens sampling formula, as described in the theory of soft selective sweeps ([7, 9]).

M.3 Allele frequency distributions

Eq (M.10) predicts the distribution of allele frequency ratios x_i at the end of the stochastic phase of the adaptive process. Typically, the Yule process will approach convergence for $n \gtrsim 100$. In a large population, this still corresponds to a small allele frequency. However, since the allele frequency ratios remain constant also during the deterministic phase, we can use the Yule process result to derive the distribution of mutant allele frequencies also at a later stage, when (partial or complete) phenotypic adaptation has been achieved. As above, we characterize the time of observation via the frequency of the ancestral phenotypes f_w that is still found in the population. We treat the case of full adaptation, $f_w = 0$, before we turn to the case of a general f_w.

Complete phenotypic adaptation, $f_w = 0$

If selection is very strong, complete fixation of the mutant phenotype may be rapidly achieved. For any non-zero level of recombination among loci, $f_w = 0$ requires, in our model, that there is (at least) a single locus where the mutant allele has reached fixation. In the following, we will call the locus with the largest mutant frequency the *major locus* and all other loci *minor loci*. We are interested in the joint distribution of allele frequencies when the major locus has reached fixation. From (M.10), we can derive the probability that the first locus ends up being the major locus as

$$\mathrm{P}_{1>}^{(\boldsymbol{\Theta})} = \int_0^1 \cdots \int_0^1 \mathrm{P}_{\mathrm{inDir}}[\{x_i\}_{i\geq 2}|\boldsymbol{\Theta}]\, dx_2 \dots dx_L\,. \tag{M.17}$$

Since allele frequencies p_i equal allele frequency ratios x_i relative to the major locus in this case, the joint distribution at all minor loci, $\{p_i\}_{i\geq 2}$, $0 \leq p_i \leq 1$, conditioned on fixation of the mutant allele at the first locus, follows as $\mathrm{P}_{\mathrm{inDir}}[\{p_i\}_{i\geq 2}|\boldsymbol{\Theta}]/\mathrm{P}_{1>}[\boldsymbol{\Theta}]$. The joint allele frequency distribution for all loci at $f_w = 0$ results as product of a Dirac point measure at the major locus and truncated inverted Dirichlet densities at the minor loci. Summing over all possible loci as major locus we obtain

$$\mathrm{P}_0[\{p_i\}_{i\geq 1}|\boldsymbol{\Theta}] = \sum_{k=1}^{L} \left(\frac{\delta_{p_k - 1}}{B[\boldsymbol{\Theta}]} \prod_{j\neq k} p_j^{\Theta_j - 1} \Big(1 + \sum_{j\neq k} p_j\Big)^{-\sum_{j=1}^{L}\Theta_j} \right), \tag{M.18}$$

where the Dirac δ constrains the distribution to the boundary faces $p_k = 1$ of the L-dimensional hypercube $[0,1]^L$ of allele frequencies.

Note that this formula is independent of linkage patterns as long as loci can recombine at all and are not completely linked (see below for this case).

Incomplete phenotypic adaptation, $f_w > 0$, linkage equilibrium

While the distribution of allele frequency *ratios* x_i, Eq (M.10), holds for any time of observation during the adaptive process (once the Yule process has reached convergence), the corresponding distribution (M.18) for the *absolute* allele frequencies p_i holds only for complete phenotypic adaptation, $f_w = 0$. To derive this distribution for arbitrary $f_w \geq 0$, we need to translate the stopping condition for the ancestral phenotype to a condition on the p_i. For $f_w = 0$, this just leads to the condition $p_k = 1$ for the major locus, constraining the distribution (M.18) to the boundary faces of the allele frequency hypercube. Importantly, this constraint is independent of linkage. For $f_w > 0$, in contrast, any constraint on the distribution of the p_i due to the stopping condition will necessarily also depend on the linkage disequilibria. For further analytical progress we now assume that recombination is sufficiently strong that linkage disequilibria can be ignored. We then obtain

$$\prod_{j=1}^{L}(1-p_j) = f_w \tag{M.19}$$

and the joint allele frequency distribution is given by the following Theorem, which is our main analytical result.

Theorem 2 If the adaptive process is stopped at a frequency f_w of the ancestral phenotype in the population, and assuming linkage equilibrium among loci, the joint distribution of mutant frequencies on the L-dimensional hypercube is

$$\mathrm{P}_{f_w}[\{p_i\}_{i\geq 1}|\mathbf{\Theta}] = \frac{\delta_{\prod_{j=1}^{L}(1-p_j)-f_w}}{B[\mathbf{\Theta}]} \prod_{i=1}^{L} p_i^{\Theta_i - 1} \left(\sum_{j=1}^{L} p_j\right)^{-\sum_{j=1}^{L}\Theta_j} \left(\sum_{j=1}^{L} \frac{f_w\, p_j}{1-p_j}\right), \tag{M.20}$$

where the δ-function restricts the support of $\mathrm{P}_{f_w}[\{p_i\}_{i\geq 1}|\mathbf{\Theta}]$ to the $(L-1)$-dimensional submanifold $\prod_{j=1}^{L}(1-p_j) = f_w$.

Proof We can rewrite (M.19) as condition on the frequency p_1 at the first locus,

$$p_1 = 1 - \frac{f_w}{\prod_{j=2}^{L}(1-p_j)} \tag{M.21}$$

to obtain the transformation from frequency ratios x_i to absolute allele frequencies p_i, $i \geq 2$,

$$x_i = \frac{p_i}{p_1} = \frac{p_i \prod_{j=2}^{L}(1-p_j)}{\prod_{j=2}^{L}(1-p_j) - f_w}\,. \tag{M.22}$$

The corresponding Jacobian matrix reads $(2 \leq i, j \leq L)$

$$\tilde{\mathbf{J}}_{ij} = \frac{\partial x_i}{\partial p_j} = \frac{p_i}{1-p_j}\frac{f_w \prod_{k=2}^{L}(1-p_k)}{(\prod_{k=2}^{L}(1-p_k)-f_w)^2} + \delta_{i,j}\frac{\prod_{k=2}^{L}(1-p_k)}{\prod_{k=2}^{L}(1-p_k)-f_w}\,.$$
$$= \frac{p_i}{1-p_j}\frac{1-p_1}{p_1^2} + \frac{\delta_{i,j}}{p_1}\,.$$

Thus

$$\tilde{\mathbf{J}} = \frac{1-p_1}{p_1^2}\,\mathbf{Q} + \frac{1}{p_1}\,\mathbf{I}\,,$$

where $\mathbf{I}$ is the identity matrix and $\mathbf{Q}_{i,j} = p_i/(1-p_j)$. Since $\mathbf{Q}$ has the eigenvalue $\sum_j p_j/(1-p_j)$ and a $(L-2)$-fold eigenvalue 0, we obtain the spectrum of $\tilde{\mathbf{J}}$ and thus the determinant

$$\mathrm{Det}[\tilde{\mathbf{J}}] = p_1^{1-L}\left(\sum_{j=1}^{L}\frac{p_j(1-p_1)}{(1-p_j)p_1}\right). \tag{M.23}$$

From (M.10), we then obtain the joint distribution of locus frequencies $p_2, \ldots, p_L$ at the stopping condition (M.21) as

$$\mathrm{P}_{f_w}[\{p_i\}_{i\geq 2}|\mathbf{\Theta}] = \frac{\mathrm{Det}[\tilde{\mathbf{J}}]}{B[\mathbf{\Theta}]}\prod_{i=2}^{L}\left(\frac{p_i}{p_1}\right)^{\Theta_i-1}\left(1+\sum_{j=2}^{L}\frac{p_j}{p_1}\right)^{-\sum_{j=1}^{L}\Theta_j}$$
$$= \frac{1}{B[\mathbf{\Theta}]}\prod_{i=1}^{L}p_i^{\Theta_i-1}\left(\sum_{j=1}^{L}p_j\right)^{-\sum_{j=1}^{L}\Theta_j}\left(\sum_{j=1}^{L}\frac{p_j(1-p_1)}{1-p_j}\right) \tag{M.24}$$

where the dependence on f_w is implicit in $p_1 = p_1(f_w)$, as given in (M.21). The joint distribution over all L loci follows as

$$\mathrm{P}_{f_w}[\{p_i\}_{i\geq 1}|\mathbf{\Theta}] = \delta_{p_1-1+f_w/\prod_{j=2}^{L}(1-p_j)}\,\mathrm{P}_{f_w}[\{p_i\}_{i\geq 2}|\mathbf{\Theta}]\,. \tag{M.25}$$

Note that we do not assume that the first locus is the major locus in (M.25). Finally, the symmetrical form (M.20) results from the relation

$$\delta_{g(x)-c} = \frac{\delta_{x-x_c}}{|g'(x)|_{x_c}|} \quad ; \quad g(x_c) = c$$

for the Dirac δ-function.

Remarks

1. To obtain marginal distributions for single loci we generally need to perform a $(L-2)$-dimensional integral (after resolving the δ-function). Details for specific cases used in the main part of the article are provided in the Mathematica notebook. For two loci, simple explicit formulas for marginal distributions can be derived. E.g., the marginal distribution at the first locus reads

$$\mathrm{P}_{f_w}[p_1|\Theta_1,\Theta_2] = \frac{p_1^{\Theta_1-1}(1-p_1-f_w)^{\Theta_2-1}(1-p_1)^{\Theta_1+1}}{B[\Theta_1,\Theta_2]\,(1-p_1^2-f_w)^{\Theta_1+\Theta_2}}\left(1-\frac{f_w(1-2p_1)}{(1-p_1)^2}\right) \tag{M.26}$$

for $0 \leq p_1 \leq f_w$. The distribution has singularities at $p_1 = 0$ for $\Theta_1 < 1$ and at $p_1 = 1 - f_w$ for $\Theta_2 < 1$. The distributions $\mathrm{P}^{+}_{f_w}[p|\Theta_1,\Theta_2]$ at the major locus and $\mathrm{P}^{-}_{f_w}[p|\Theta_1,\Theta_2]$ at the minor locus (which can either be locus 1 or locus 2) follow as

$$\mathrm{P}^{\pm}_{f_w}[p|\Theta_1,\Theta_2] = \big(\mathrm{P}_{f_w}[p|\Theta_1,\Theta_2] + \mathrm{P}_{f_w}[p|\Theta_2,\Theta_1]\big)\, H_{\pm(p-1+\sqrt{f_w})} \tag{M.27}$$

where $H(x)$ is the Heaviside function with $H_x = 1$ for $x \geq 0$ and $H_x = 0$ else. Finally, the *conditioned* distributions $\mathrm{P}^{1\gtrless}_{f_w}[p_1|\Theta_1,\Theta_2]$ at the first locus if this locus is the major/minor locus are

$$\mathrm{P}^{1>}_{f_w}[p_1|\Theta_1,\Theta_2] = \frac{\mathrm{P}_{f_w}[p_1|\Theta_1,\Theta_2]}{\mathrm{P}^{(\Theta_1,\Theta_2)}_{1>}}\, H_{p_1-1+\sqrt{f_w}}\,, \tag{M.28a}$$

$$\mathrm{P}^{1<}_{f_w}[p_1|\Theta_1,\Theta_2] = \frac{\mathrm{P}_{f_w}[p_1|\Theta_1,\Theta_2]}{1-\mathrm{P}^{(\Theta_1.\Theta_2)}_{1>}}\, H_{-(p_1-1+\sqrt{f_w})}\,, \tag{M.28b}$$

where $\mathrm{P}^{(\Theta_1,\Theta_2)}_{1>}$, defined in Eq (M.17), evaluates to a Hypergeometric function for general $\Theta_1 \neq \Theta_2$, but reduces to 1/2 for $\Theta_1 = \Theta_2$.

2. The marginal distribution for p_k has a singularity at $p_k = 0$ for $\Theta_k < 1$ and a singularity at $p_k = 1 - f_w$ for $\sum_{j\neq k}^{L} \Theta_j < 1$. To see this, consider the marginal distribution of p_L, which is obtained from Eq. (M.25) after integartion over $p_1, \ldots, p_{L-1}$. Dropping non-singular terms (such as the sums in Eq M.24), and defining

$$q_k = \frac{\prod_{j=k+1}^{L}(1-p_j) - f_w}{\prod_{j=k+1}^{L}(1-p_j)}$$

the singlular part can be written as

$$\begin{aligned}\mathrm{P}_{f_w}[p_L|\boldsymbol{\Theta}] &\sim \int_0^1\int_0^1\cdots\int_0^1 \delta_{p_1-q_1}\prod_{i=1}^{L} p_i^{\Theta_i-1}dp_1\ldots dp_{L-1}\\ &= \int_0^{q_{L-1}}\int_0^{q_{L-2}}\cdots\int_0^{q_2} q_1^{\Theta_1-1}\prod_{i=2}^{L} p_i^{\Theta_i-1}dp_2\ldots dp_{L-1}\,,\end{aligned}$$

after performing the p_1 integral. The upper integral limits q_k account for the constraint $q_1 > 0$. Substituting

$$\tilde{p}_2 := \frac{p_2}{q_2} \quad \Rightarrow \quad dp_2 = q_2\, d\tilde{p}_2$$

and using that $q_1 = q_2(1-\tilde{p}_2)/(1-\tilde{p}q_2)$ we obtain

$$\mathrm{P}_{f_w}[p_L|\boldsymbol{\Theta}] \sim \int_0^{q_{L-1}} \cdots \int_0^{q_3} \int_0^1 q_1^{\Theta_1-1} q_2^{\Theta_2} \tilde{p}_2^{\Theta_2-1} \prod_{i=3}^{L} p_i^{\Theta_i-1} d\tilde{p}_2 dp_3 \ldots dp_{L-1}$$

$$= \int_0^{q_{L-1}} \cdots \int_0^{q_3} q_2^{\Theta_1+\Theta_2-1} \int_0^1 \left(\frac{1-\tilde{p}_2}{1-\tilde{p}_2 q_2}\right)^{\Theta_1-1} \tilde{p}_2^{\Theta_2-1} d\tilde{p}_2 \prod_{i=3}^{L} p_i^{\Theta_i-1} dp_3 \ldots dp_{L-1}.$$

Since the $\tilde{p}_2$ integral is bounded by $1/\Theta_2$ from below and by $1/\Theta_2 + 1/\Theta_1$ from above for all $0 \le q_2 \le 1$, it does not contribute to a singularity in $\mathrm{P}_{f_w}[p_L|\boldsymbol{\Theta}]$. For the singular part, we thus have

$$\mathrm{P}_{f_w}[p_L|\boldsymbol{\Theta}] \sim \int_0^{q_{L-1}} \cdots \int_0^{q_3} q_2^{\Theta_1+\Theta_2-1} \prod_{i=3}^{L} p_i^{\Theta_i-1} dp_3 \ldots dp_{L-1}.$$

Iterating the substitution procedure for variables p_3 to p_{L-1}, we arrive at

$$\mathrm{P}_{f_w}[p_L|\boldsymbol{\Theta}] \sim q_{L-1}^{\sum_{j=1}^{L-1}\Theta_j - 1} p_L^{\Theta_L-1} = \left(\frac{1-f_w-p_L}{1-p_L}\right)^{\sum_{j=1}^{L-1}\Theta_j-1} p_L^{\Theta_L-1},$$

demonstrating the singular behavior for $p_L \to 0$ and for $p_L \to 1-f_w$. Since the labeling of loci is arbitrary, the assertion follows for all loci.

Incomplete phenotypic adaptation, $f_w > 0$, tight linkage

Even if all loci are completely linked, the joint distribution of allele frequency *ratios* is still given by (M.10). However, the transformation to absolute allele frequencies at the stopping condition $f_w \neq 0$ depends on linkage. Because all mutant alleles are rare during the stochastic phase, we can ignore haplotypes with more than a single mutant during this time. Since we ignore new mutations during the deterministic phase, mutant alleles stay in maximal linkage disequilibrium in the absence of recombination. We thus have

$$\sum_{j=1}^{L} p_j = 1 - f_w \quad \Rightarrow \quad x_i = \frac{p_i}{p_1} = \frac{p_i}{1-f_w-\sum_{j=2}^{L} p_j}$$

with corresponding Jacobian

$$\mathbf{J}_{ij} = \frac{\partial x_i}{\partial p_j} = \frac{p_i + \delta_{i,j}\, p_1}{p_1^2} \quad ; \quad \mathrm{Det}[\mathbf{J}] = \frac{1-f_w}{p_1^L}.$$

Using this transformation on (M.10), the joint distribution of mutant frequencies reads

$$\mathrm{P}_{f_w,\mathrm{tl}}[\{p_i\}_{i\geq 1}|\boldsymbol{\Theta}] = \frac{\delta_{\sum_{i=1}^{L} p_i - 1 + f_w}}{B[\boldsymbol{\Theta}](1-f_w)^{L-1}} \prod_{i=1}^{L} \left(\frac{p_i}{1-f_w}\right)^{\Theta_i - 1} . \tag{M.29}$$

Evidently, this is just the Dirichlet distribution on the cube $[0, 1-f_w]^L$. This is expected since the problem reduces to a single-locus, L-alleles problem for tight linkage. The marginal distributions can be derived for an arbitrary number of loci and are given by transformed β-distributions,

$$\mathrm{P}_{f_w,\mathrm{tl}}[p_k|\boldsymbol{\Theta}] = \frac{(1-f_w)^{-1}}{B[\boldsymbol{\Theta}]} \left(\frac{p_k}{1-f_w}\right)^{\Theta_k - 1} \left(1 - \frac{p_k}{1-f_w}\right)^{\left(\sum_{j\neq k}^{d} \Theta_j\right) - 1} , \tag{M.30}$$

with singularities at the boundaries $p_k = 0$ for $\Theta_k < 1$ and at $p_k = 1 - f_w$ for $\sum_{j\neq k} \Theta_j < 1$ as in the linkage equilibrium case. For two tightly linked loci, the major locus must have frequency $p > (1-f_w)/2$. The distribution at the major/minor locus therefore reads

$$\mathrm{P}^{\pm}_{f_w,\mathrm{tl}}[p|\Theta_1, \Theta_2] = \left(\mathrm{P}_{f_w,\mathrm{tl}}[p|\Theta_1, \Theta_2] + \mathrm{P}_{f_w,\mathrm{tl}}[p|\Theta_2, \Theta_1]\right) H_{\pm(p-(1-f_w)/2)} \tag{M.31}$$

and conditioned distributions follow as in (M.28).

References

1. Hermisson J, Pennings PS. Soft sweeps: Molecular population genetics of adaptation from standing genetic variation. Genetics. 2005;169(4):2335–2352.

2. Joyce P, Tavaré S. Cycles, permutations and the structure of the Yule process with immigration. Stochastic processes and their applications. 1987;25:309–314.

3. Durrett R. Probability: Theory and Examples. Cambridge University Press; 2010.

4. Etheridge A, Pfaffelhuber P, Wakolbinger A, An approximate sampling formula under genetic hitchhiking. The Annals of Applied Probability. 2006;16(2):685–729.

5. Hermisson J, Pfaffelhuber P. The pattern of genetic hitchhiking under recurrent mutation. Electronic Journal of Probability. 2008;13:2069–2106.

6. Uecker H, Hermisson J. On the fixation process of a beneficial mutation in a variable environment. Genetics. 2011;188(4):915–930.

7. Pennings PS, Hermisson J. Soft sweeps II: Molecular population genetics of adaptation from recurrent mutation or migration. Molecular Biology and Evolution. 2006;23(5):1076–1084.

8. Wright S. Evolution in Mendelian populations. Genetics. 1931;16(2):97–159.

9. Hermisson J, Pennings PS. Soft sweeps and beyond: Understanding the patterns and probabilities of selection footprints under rapid adaptation. Methods in Ecology and Evolution. 2017;8(6):700–716.
