# Supplementary material for: Polygenic adaptation: From sweeps to subtle frequency shifts
Source: PLoS Genet. 2019 Mar 20;15(3):e1008035. doi: 10.1371/journal.pgen.1008035 (PMC6443195; doi:10.1371/journal.pgen.1008035)
Supplement: S1 Fig — (PDF) [file pgen.1008035.s003.pdf]

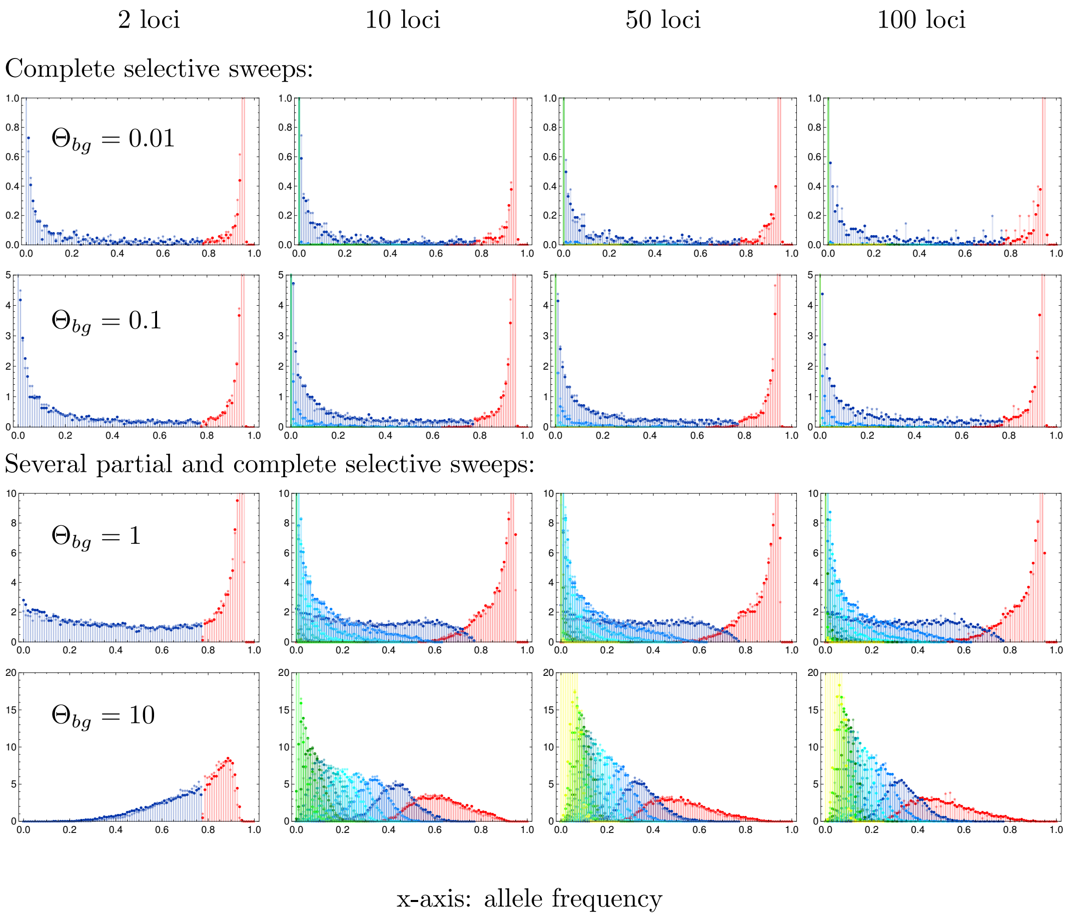

Figure S1: Genetic architecture with weak selection. Frequency distributions of major and minor loci are shown upon an increase of 95% in mean fitness for complete redundancy for $s_b = 0.1$ (colored dots, data as in main text Fig 4) and weaker selection $s_b = 0.01$ (colored asterisks). Deleterious selection before the environmental change is set to $s_d = -s_b$. As we condition on adaptation from the ancestral state, we do not obtain enough valid runs for $s_d = -0.01$ and $\Theta_{bg} = 100$.
