# Supplementary material for: Polygenic adaptation: From sweeps to subtle frequency shifts
Source: PLoS Genet. 2019 Mar 20;15(3):e1008035. doi: 10.1371/journal.pgen.1008035 (PMC6443195; doi:10.1371/journal.pgen.1008035)
Supplement: S2 Fig — (PDF) [file pgen.1008035.s004.pdf]

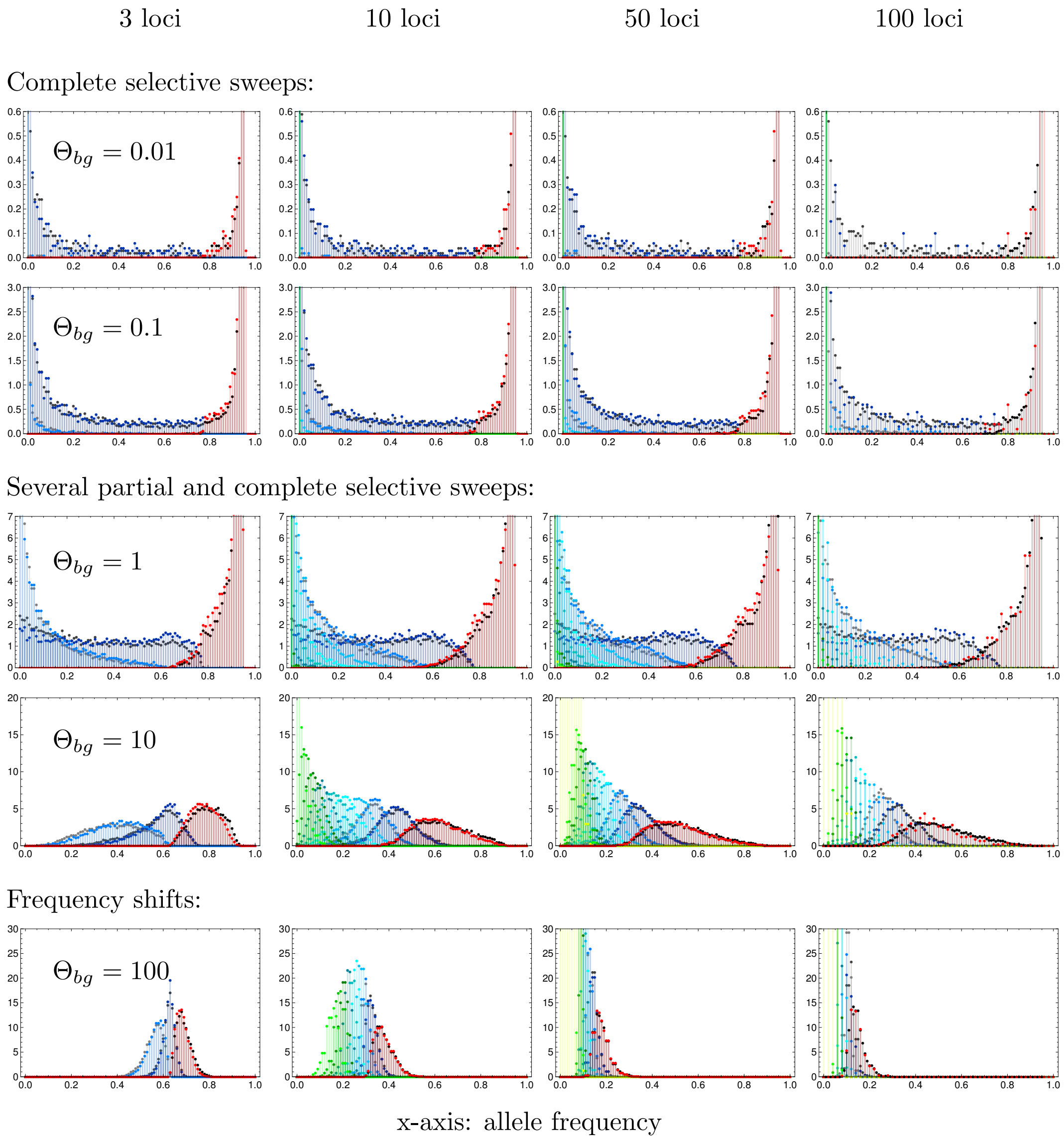

Figure S2: Weakly relaxed redundancy. Weakly relaxing redundancy such that a single mutant has fitness $1 + 0.9s_{b/d}$ and only two mutations or more confer the full fitness effect $(1 + s_{b/d})$ demonstrates the robustness of our model. As in main text Fig 4, allele frequency distributions of derived alleles are displayed once the frequency of the wildtype individuals in the population has decreased to $f_w = 5\%$, which corresponds to an increase of 95% in mean fitness for complete redundancy. Genomic patterns of adaptation show very similar characteristics as with complete redundancy. Simulation data for relaxed redundancy (colored dots) are almost identical to results for complete redundancy (gray dots).
